# Genotyping-by-sequencing markers facilitate the identification of quantitative trait loci controlling resistance to *Penicillium expansum* in *Malus sieversii*

**DOI:** 10.1371/journal.pone.0172949

**Published:** 2017-03-03

**Authors:** John L. Norelli, Michael Wisniewski, Gennaro Fazio, Erik Burchard, Benjamin Gutierrez, Elena Levin, Samir Droby

**Affiliations:** 1 Appalachian Fruit Research Station, Agricultural Research Service, United States Department of Agriculture, Kearneysville, West Virginia, United States of America; 2 Plant Genetic Resources Research, Agricultural Research Service, United States Department of Agriculture, Geneva, New York, United States of America; 3 Department of Postharvest Science, Agricultural Research Organization, the Volcani Center, Bet Dagan, Israel; Washington State University, UNITED STATES

## Abstract

Blue mold caused by *Penicillium expansum* is the most important postharvest disease of apple worldwide and results in significant financial losses. There are no defined sources of resistance to blue mold in domesticated apple. However, resistance has been described in wild *Malus sieversii* accessions, including plant introduction (PI)613981. The objective of the present study was to identify the genetic loci controlling resistance to blue mold in this accession. We describe the first quantitative trait loci (QTL) reported in the Rosaceae tribe Maleae conditioning resistance to *P*. *expansum* on genetic linkage group 3 (qM-*Pe*3.1) and linkage group 10 (qM-*Pe*10.1). These loci were identified in a *M*.*× domestica* ‘Royal Gala’ X *M*. *sieversii* PI613981 family (GMAL4593) based on blue mold lesion diameter seven days post-inoculation in mature, wounded apple fruit inoculated with *P*. *expansum*. Phenotypic analyses were conducted in 169 progeny over a four year period. PI613981 was the source of the resistance allele for qM-*Pe*3.1, a QTL with a major effect on blue mold resistance, accounting for 27.5% of the experimental variability. The QTL mapped from 67.3 to 74 cM on linkage group 3 of the GMAL4593 genetic linkage map. qM-*Pe*10.1 mapped from 73.6 to 81.8 cM on linkage group 10. It had less of an effect on resistance, accounting for 14% of the experimental variation. ‘Royal Gala’ was the primary contributor to the resistance effect of this QTL. However, resistance-associated alleles in both parents appeared to contribute to the least square mean blue mold lesion diameter in an additive manner at qM-*Pe*10.1. A GMAL4593 genetic linkage map composed of simple sequence repeats and ‘Golden Delicious’ single nucleotide polymorphism markers was able to detect qM-*Pe*10.1, but failed to detect qM-*Pe*3.1. The subsequent addition of genotyping-by-sequencing markers to the linkage map provided better coverage of the PI613981 genome on linkage group 3 and facilitated discovery of qM-*Pe*3.1. A DNA test for qM-*Pe*3.1 has been developed and is currently being evaluated for its ability to predict blue mold resistance in progeny segregating for qM-*Pe*3.1. Due to the long juvenility of apple, the availability of a DNA test to screen for the presence of qM-*Pe*3.1 at the seedling stage will greatly improve efficiency of breeding apple for blue mold resistance.

## Introduction

Blue mold of apple fruit caused by *Penicillium expansum* Link. is regarded as the most important postharvest disease of apples worldwide [1–2]. Postharvest decay of apples has been estimated to cause economic losses of over $4 million per year in the United States alone [2]. *P*. *expansum* not only causes fruit decay but also produces the carcinogenic mycotoxin, patulin, making blue mold of great concern to the food processing industry [3]. *P*. *expansum* is a necrotrophic (feeding on dead tissue) fungus, which most commonly infects fruit via wounds [4]. Mature and overripe fruit are more susceptible to infection by *P*. *expansum* than immature fruit [5–6]. Blue mold is typically controlled by treating harvested fruit with chemical fungicides prior to cold storage. Extensive research has been conducted on the biological control of blue mold and some commercial products are available [7–9]. Although domesticated apple (*Malus× domestica* Borkh.) cultivars vary in their quantitative susceptibility to blue mold, cultivars with high levels of resistance to *P*. *expansum* have not been identified [5, 10–11].

Domesticated crop cultivars inevitably represent a subset of the genetic variation found in their wild ancestors due to genetic bottlenecks that result during the process of crop domestication [12–13]. *Malus sieversii* (Lebed.) M. Roem., a wild apple species native to Central Asia, is one of the ancestral progenitors of the domesticated apple [14–16]. What distinguishes *M*. *sieversii* from other wild apple species as a desirable source for disease resistance in apple scion breeding is the unique occurrence of large, palatable fruit within the species [17]. Collaborative efforts between the United States Department of Agriculture, Agricultural Research Service (USDA-ARS), U.S. scientists, and Central Asian counterparts resulted in several successful expeditions between 1989 and 1996 to collect *M*. *sieversii* in Kazakhstan, Tajikistan, and Uzbekistan. These expeditions produced an extensive collection of over 130,000 seeds and vegetative materials collected from 44 elite accessions with phenotypes of interest [15, 18–20]. Several of these seed and elite accessions of *M*. *sieversii* were later found to be resistant to *P*. *expansum* [21–22], including USDA-ARS National Plant Germplasm System plant introduction (PI)613981 [23].

The genus *Malus* belongs to the *Maleae* tribe of the *Amygdaloideae* subfamily of the Rosaceae family and comprises many interbreeding species with 25 to 40 taxa within the genus, depending upon the system of classification [24–25]. Unlike other members of the Rosaceae, which have haploid chromosome numbers of 7, 8 or 9, members of the *Maleae* have a haploid chromosome number of 17, which appears to have been derived from a genome wide duplication of an ancient x = 9 ancestor and subsequent loss of a chromosome [16]. Genetically, *M*. *× domestica* is an allopolyploid exhibiting both monogenic and disomic inheritance from homoeologous chromosomes, but does not exhibit tetrasomic inheritance [26].

*M*.*× domestica* is a highly admixed species derived from at least four progenitor species, including *M*. *sieversii*, *M*. *orientalis* Uglitzk., *M*. *sylvestris* (L.) Mill. and *M*. *prunifolia* (Wild.) Borkh. [14,27]. Molecular analysis of 23 genes across 74 *Malus sp*. accessions identified *M*. *sieversii* as the primary progenitor of *M*.*× domestica* [16]. Analysis of chloroplast DNA, however, suggests that *M*. *× domestica* belongs to a highly admixed network of species that includes species native to China and Western North America [27]. Although these reports have established both the importance of *M*. *sieversii* as a major progenitor of *M*. *× domestica* and the importance of admixture from other species in the domestication of apple, the ancient introgressions that led to the modern apple remain uncertain. Most modern *M*. × *domestica* cultivars have been derived from a relatively small number of founder cultivars and share a high degree of genetic identity [28–29]. In contrast, wild *M*. *sieversii* collected in Central Asia displays high levels of both phenotypic and genetic diversity, and genetic studies have indicated that the diversity present in *M*. *sieversii* is greater than that of other available *Malus* sp. accessions [18,30].

Being self-incompatible, *Malus* species are usually out-crossing, hence highly heterozygous. The *Malus* genome is known to vary in both size and structure. Among 100 accessions of *Malus*, which included both *M*. × *domestica* (59 accessions) and *M*. *sieversii* (14 accessions), genome size was found to vary by approximately 15% with 2C diploid values ranging from 1.44 to 1.72 pg [31]. Copy-number variation, defined as deletions, duplications or insertions of DNA sequence fragments longer than 50 base pairs in length, have been found to be common in all 17 chromosomes of the *M*. × *domestica* genome [32].

Simple sequence repeats (SSRs) within DNA have historically played a major role in the genetic analysis of apple and remain useful because they are co-dominant, highly polymorphic, abundant and reliably reproducible [33–35]. The development of a whole genome sequence for ‘Golden Delicious’ apple in 2010, and the subsequent development of genotyping arrays based upon that sequence, have greatly aided and advanced genetic analysis of *M*. × *domestica* [16,36–39]. Previous evaluation of *M*. × *domestica* derived single nucleotide polymorphism (SNP) markers suggested that such markers derived from the ‘Golden Delicious’ genome would also be useful for the genetic analysis of *M*. *sieversii* [40–41].

To date, the application of next generation sequencing to genetic mapping in apple has been more limited [42–43]. Genotyping-by-sequencing (GBS) markers are SNPs identified from direct sequencing of genomic libraries constructed by using methylation-sensitive restriction enzymes that target low copy regions of the genome with two-to-three higher efficiency [44]. The GBS approach is cost effective in comparison to other genotyping methods and capable of reaching regions of the genome that may be inaccessible to genotyping by sequence capture arrays.

The hypothesis tested in the present study was that the observed resistance of PI613981 to infection by *P*. *expansum* is due to the effect of specific genetic loci. To test this hypothesis, a *M*.*× domestica* ‘Royal Gala’ X *M*. *sieversii* PI613981 full-sib family, designated GMAL4593, was phenotyped for blue mold resistance, genotyped to develop a genetic linkage map, and then analyzed for the presence of QTLs associated with resistance. Using a genetic linkage map developed from SSR and *M*. *× domestica* SNP markers, a QTL associated with resistance to *P*. *expansum* was found on linkage group (LG) 10. However, its calculated logarithm of likelihood odds (LOD) did not seem to fully account for the observed differences in resistance among the GMAL4593 progeny. Subsequent addition of GBS markers to the genetic linkage map facilitated the identification of a QTL on LG3 associated with resistance to *P*. *expansum* that was of far greater magnitude.

## Results

### Mapping population GMAL4593 segregated for resistance to *P*. *expansum*

Since the GMAL4593 population was known to segregate for fruit maturity date based upon previous field observations, fruit used in the current study was harvested over multiple harvest dates (Table 1). Within a given calendar year, most accessions were harvested at a single harvest date, however, some accessions with sufficient fruit were harvested on more than one date. Due to both the biennial and sporadic nature of fruit bearing within the GMAL4593 mapping population, progeny were evaluated for resistance to blue mold over a four year period with 19, 35, 55, and 52 progeny evaluated for a total of 1, 2, 3 or 4 years, respectively, generating an average of 2.7 trial years per progeny (Table 1). Eight accessions of the mapping population were not evaluated for resistance because a sufficient number of fruit was not available in any of the years. In 2011 and 2014, fruit of the GMAL4593 population ripened over a 6.5 week period. To accommodate the extended harvest period and insure complete postharvest maturation, fruit were evaluated for blue mold resistance several times during each harvest season (Table 1). Drought conditions were severe in 2012, with 42% less precipitation than the average seasonal precipitation of the other three years, and fruit ripening in this year was compressed to a 13 d period (Table 1). The overall mean fruit weight in the GMAL4593 population was lower in 2012, while fruit firmness and soluble solids were higher, and starch content and titrateable acidity were unaffected, relative to the other years (S1 Table).

**Table 1:** Summary of apple fruit harvested from progeny of the GMAL4593 mapping population and used for the evaluation of resistance to blue mold postharvest decay.

Year	Total # progeny evaluated	Seasonal precipitation (cm)	Harvest interval	Total number of harvest dates	Number of blue mold tests
2011	134	47.8	24 Aug– 3 Oct	10	3
2012	110	22.4	23 Aug– 4 Sep	3	4
2013	102	51.3	22 Aug– 11 Sep	6	5
2014	116	60.5	25 Aug– 1 Oct1	7	3

Trees were grown on their own roots (seedlings) without irrigation at the USDA-ARS, Plant Genetics Resources Research, Geneva, New York. The planting contained 181 individuals. However, only 171 progeny were used to construct genetic linkage maps due to both the occurrence of out-crossing and culling due to poor or missing genotype data. Seasonal precipitation was the total precipitation from May 1 to September 30. Harvest interval was defined by the dates of the first and last fruit harvests.

The measure of host resistance to blue mold used in the present study was the diameter of blue mold lesions 7 days post-inoculation (dpi) in uniform fruit surface wounds inoculated with *P*. *expansum*. Because fruit maturity and firmness are known to affect susceptibility of fruit to blue mold [5–6], both fruit starch content and firmness were initially used as co-factors in calculating least square means (LSmean) of lesion diameter 7 dpi. In all four years, both genotype (progeny accession) and fruit starch content were determined to have significant (*P*<0.05) effects on blue mold lesion diameter 7 dpi, whereas fruit firmness did not (Table 2). Therefore, resistance was quantified by LSmean of lesion diameter 7 dpi calculated from a mixed linear model in which accession and fruit starch content were fixed-effects parameters, and fruit harvest date and the specific blue mold evaluation test were random-effects parameters (SAS Mixed Procedure, SAS Institute Inc., Cary, NC). When combining data over multiple years, year was also included as a fixed-effects parameter (*P* = 0.0068 for all years, and *P* = 0.0052 when the drought year 2012 was not included).

**Table 2:** Probability (*P)* that genotype (progeny), fruit firmness, and fruit starch content had no effect on blue mold lesion diameter 7 days post-inoculation.

Year	Genotype	Firmness	Starch Content
2011	<0.0001	0.7134	0.0003
2012	<0.0001	0.8265	<0.0001
2013	<0.0001	0.1034	0.0131
2014	<0.0001	0.2515	0.0006

*P* values based on F-value calculated from a mixed linear model in which fruit harvest date and blue mold evaluation test were random-effect parameters.

**Fig 1:**
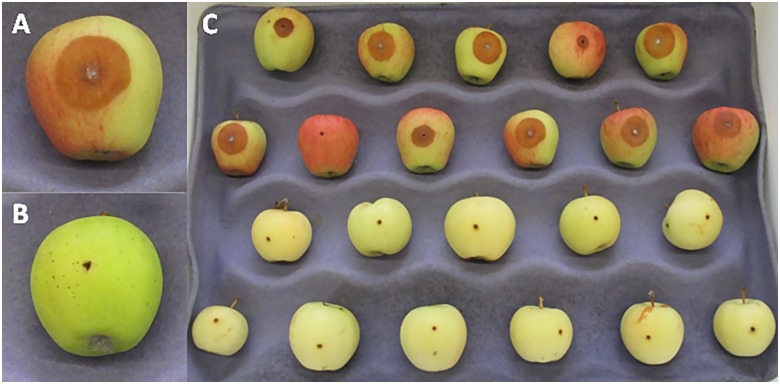
Blue mold (*Penicillium expansum*) lesions on wounded, mature apple fruit of susceptible and resistant individuals in the GMAL4593 population at 7 days post-inoculation (dpi). **A:** susceptible response to infection by *P*. *expansum*, **B**: highly resistant response to *P*. *expansum*, and **C:** in each test 11 replicate fruit of each progeny were inoculated and evaluated at 7 dpi.

**Fig 2:**
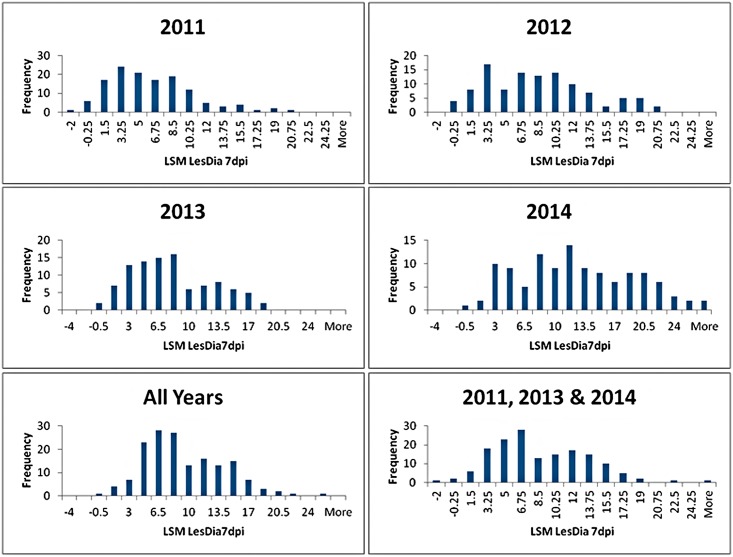
Histograms illustrating the frequency distribution of blue mold resistance within the GMAL4593 mapping population in multiple years. Resistance measure was the least square (LS)mean of blue mold lesion diameter 7 days post-inoculation (LSM LesDia 7dpi in figure) of a uniform, fruit-surface wound with *P*. *expansum* calculated from a mixed linear model in which accession and fruit starch content were fixed-effects parameters, and fruit harvest date and specific blue mold evaluation test were random-effects parameters.

In every evaluation of resistance there were examples of GMAL4593 progeny that were highly resistant and highly susceptible to blue mold 7dpi (Fig 1), however, there was also a quantitative gradation of resistance responses among the progeny. Segregation of the GMAL4593 population into distinct bimodal, blue mold resistant and susceptible populations was most evident in 2013, the combined data from all years, and when the 2012 drought year data was removed from all years (Fig 2).

### Adding GBS markers for a more saturated genetic linkage map of the *Malus sieversii* PI613981 genome

#### Genetic linkage map based on SSR, SNPlex, and high-resolution-melting SNP markers

A genetic linkage map was initially constructed for the GMAL4593 mapping population using SSR [33,45] and SNP markers identified in the ‘Golden Delicious’ whole genome reference sequence [16]. ‘Royal Gala’ had a significantly (*χ*^*2*^ = 103.5, *P*<0.001) greater number of informative ‘Golden Delicious’ SNP markers, identified by SNPlex technology [46–47], relative to the number of informative markers in PI613981 (Table 3). No significant difference (*χ*^*2*^ = 0.36, *P* = 0.55) in the number of SSR informative markers were observed between ‘Royal Gala’ and PI613981. The genetic linkage maps derived from these markers were found to contain multiple large gaps in the map of the PI613981 parent, which prevented assembly of some PI613981 LGs (data not shown).

**Table 3:** Number of DNA markers screened and found to be informative for the ‘Royal Gala’ and *M*. *sieversii* PI613981 parents of the GMAL4593 mapping population.

Marker Type	Number Screened	‘Gala’	PI613981	*P*
SSR	232	94	86	0.55
SNPlex	399	252	89	<0.001
SNP HRM	104	25	36	0.16
SNP GBS	NA	861	561	<0.001

A Chi-square statistic was used to determine if the null hypothesis that the number of informative markers was the same in both parents could be rejected. *P* = the probability that the number of informative markers are the same.

**Fig 3:**
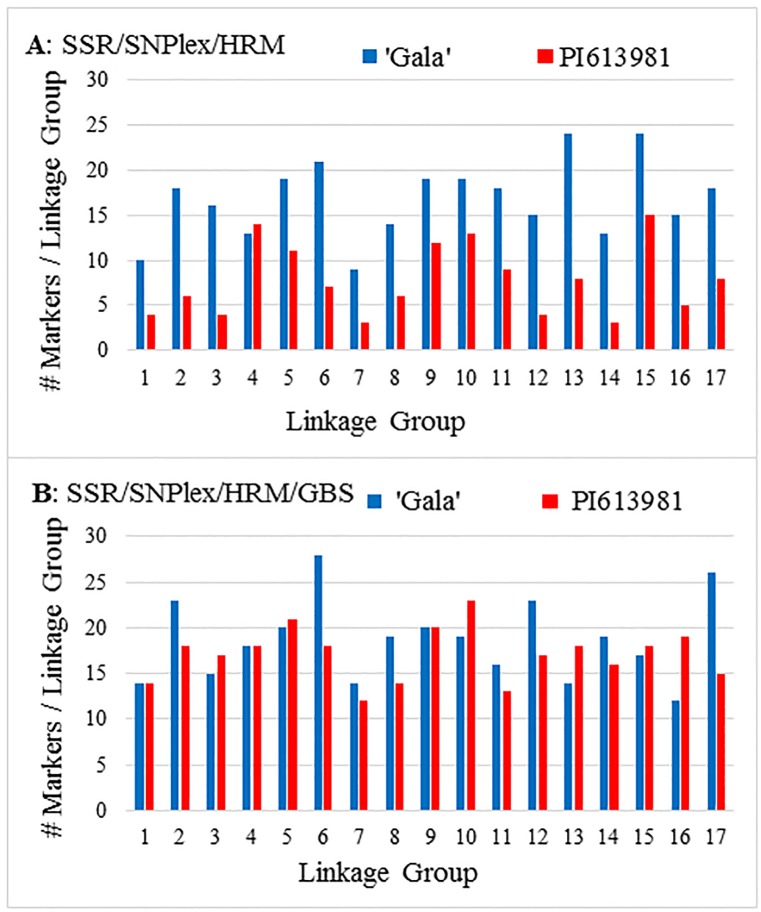
Number of markers per linkage group in ‘Royal Gala’ (blue) and PI613981 (red) genetic linkage maps derived from the GMAL4593 population when maps were composed of A) SSR, SNPlex, and HRM markers and B) SSR, SNPlex, HRM, and GBS markers.

Based upon alignment of PI613981 re-sequence data aligned to the ‘Golden Delicious’ v.1 sequence, a total of 203 DNA primer pairs were designed around 104 targeted loci for HRM analysis [48]. The HRM analysis identified 20 SNP and 16 INDELS markers that were informative within the population (35% success rate) for either PI613981 or both parents (Table 3). The additional markers and subsequent linkage analysis identified 17 LGs for both parents. However, over 40 small gaps (>10 cM) were present in the maps of both parents and 13 large gaps (>20 cM) were present in the map of PI613981 (Table 4). Marker coverage of PI613981 LG1, LG3, LG7, LG12, LG14 and LG16 remained especially poor relative to ‘Royal Gala’ (Fig 3A). Although the resulting GMAL4593 map was successfully used for the QTL analysis of some traits (data not shown), it failed to identify large effect QTLs associated with blue mold resistance (see below).

**Table 4:** Marker type, density, and coverage of the various genetic linkage maps developed for the GMAL4593 bi-parental mating population.

Genetic Map	#SSR	# SNPlex SNP	#HRMSNP	#GBSSNP	Total length(cM)	AveragecM^-1^marker	# gaps>10cM	# gaps>20cM	Largest gap(cM)
‘Royal Gala’/no GBS	64	196	21	0	1441.0	5.1	43	3	28.7
PI613981/no GBS	58	40	24	0	1091.9	9.0	41	13	45.4
GMAL4593/no GBS	91	224	27	0	1472.4	4.3	37	3	31.0
‘Royal Gala’/with GBS	51	24	15	212	1230.2	4.1	21	1	27.5
PI613981/with GBS	47	9	12	206	1722.9	6.3	50	6	29.6
GMAL4593/with GBS	69	32	20	419	1552.8	2.9	12	1	20.2

The first maternal (‘Royal Gala’), paternal (PI613981) and combined population maps were constructed using 171 individuals of the population. Because GBS data was not available for 2 of the 171 mapped individuals, maps containing GBS data (2^nd^ group of three maps) were constructed using 169 individuals.

#### Genetic linkage map based on SSR, SNPlex, HRM SNP, and GBS markers

Similar to the SNPlex markers, a significantly (*χ*^*2*^ = 47.2, *P*<0.001) greater number of GBS markers were identified for ‘Royal Gala’ compared to PI613981 (Table 3). GBS markers were combined with the SSR, SNPlex, and HRM data, and new linkage maps were constructed with markers selected at approximately 5 cM intervals to facilitate subsequent QTL analysis (Table 4, S1 Text (maps), S2 Text (genotypes), S1 Fig (maps)). Addition of the GBS markers to the PI613981 linkage map brought it to near parity with the ‘Royal Gala’ map based upon the number of markers per LG (Fig 3B). Inclusion of the GBS markers also decreased the average distance between markers for the maternal, paternal, and population linkage maps (Table 4). The total numbers of markers identified for the PI613981 map containing GBS markers more than doubled (122 to 274), whereas in the ‘Royal Gala’ map the number of markers increased only 7.5% (281 to 302). The slight change for ‘Royal Gala’ was due to the removal of SNPlex markers with missing data from the map (see Materials and methods). These were far more numerous in the initial ‘Royal Gala’ map than in the PI613981 map (169 versus 36), whereas the numbers of GBS markers was similar in both parental maps (Table 4). The number of both small and large gaps decreased by 50% or more in all three maps containing GBS markers, except for small gaps in the PI613981 map, which showed an increase primarily due to the inflation of the total PI613981 map length from 1091.9 cM to 1722.9 cM.

### QTLs for resistance to *P*. *expansum* in the GMAL4593 mapping population

**Fig 4:**
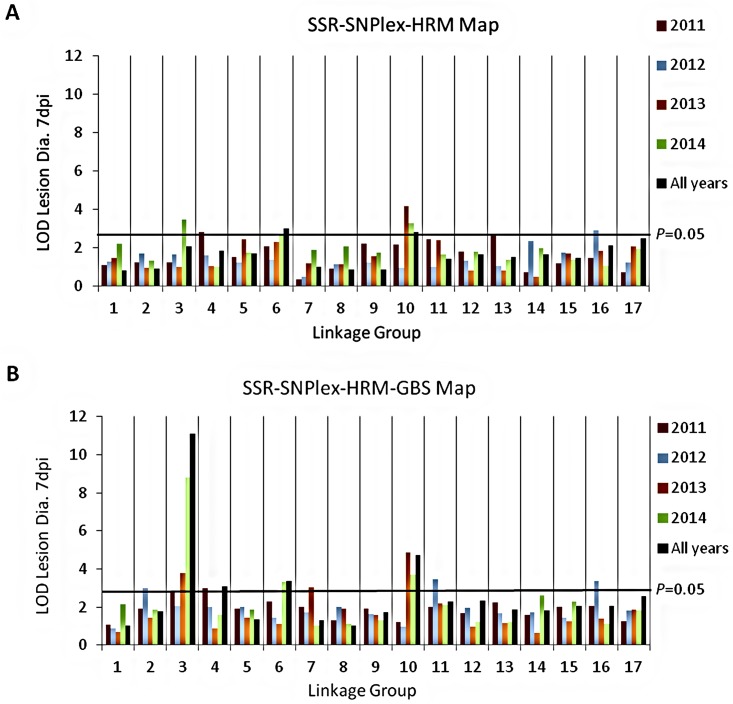
The highest interval mapping LOD score observed on an individual genetic linkage group for the association between a DNA marker interval and the LSmean of blue mold lesion diameter 7 dpi determined for each year and data combined for all four years (All years). Interval mapping LOD scores were determined using MapQTL6 (Kyazma B.V., Wageningen, the Netherlands) and genetic linkage maps for the GMAL4593 mapping population containing **A**: SSR, SNPlex, and HRM markers and **B**: SSR, SNPlex, HRM, and GBS markers. The *P* = 0.05 threshold was estimated based upon previous large-scale simulations [52].

Using the GMAL4593 genetic linkage map containing only SSR, SNPlex, and HRM markers for the analyses, interval mapping (IM) [49] identified significant associations between resistance and markers on LG10 in 2013, 2014 and all years (Fig 4A). Because LG10 contains several QTLs associated with fruit firmness and ripening [50] and ‘Royal Gala’ was the primary contributor to the resistance on LG10 (see below), it was unclear if this association with blue mold resistance was due to specific resistance factors or was rather an artifact of the effect of fruit ripening on resistance. Apart from the association with markers on LG10, the results were generally inconsistent between years and no large effect QTLs controlling resistance to blue mold could be identified. For example, in 2011 a significant QTL was identified only on LG4 and was the initial focus of our analysis [51], however in subsequent years a significant association was no longer identified on this LG using the SSR-SNPlex-HRM map (Fig 4A).

The Kruskal-Wallis test (KW) [53] and IM analysis of the same blue mold lesion diameter data using the GMAL4593 linkage map containing SSR, SNPlex, HRM, and GBS markers identified highly significant (*P=* 0.05) associations between resistance to blue mold and markers on LG3 in all of the datasets except 2012 (Fig 4B). In 2011 and 2014, the greatest KW K and IM LOD scores were positioned at 74.7 cM (marker S3_31564599), whereas in 2013 and all years the highest K and LOD scores occurred at 70.2 cM (S3_30831583). In addition to LG3, significant (*P=* 0.05) associations with resistance were identified in two or more data sets on LG4, LG6 and LG10, although at much lower K and LOD scores than the association observed on LG3 (Fig 4B). The greatest K and LOD scores on LG10 in 2013, 2014, and all years were associated with marker S10_29121625 at 73.8 cM. On LG6, the greatest LOD scores in 2014 and all years occurred at 50.5 cM (S6_22041588), whereas the greatest K scores in both data sets were with marker S6_19787309 at 38.7 cM. On LG4 the positioning of the greatest K and LOD scores were variable: in 2011 both were associated with marker S4_13073191 at 25.4 cM, whereas in all years the greatest K value was associated with marker S4_25013026 (60 cM) and the greatest LOD score was obtained at 47.7 cM (S4_23243656). A significant LOD score was also obtained on LG7 at 74.0 cM (S7_24339170) but only in 2013. In the drought year 2012, significant LOD scores occurred on LG2 at 51.9 cM (S2_17667008), on LG11 at 0.0 cM (S11_557388), and on LG16 at 5.3 cM (Ch02d10a). These associations were not observed, however, in any other individual year or all years. Furthermore, no significant associations were observed on LG3, LG4, LG6 or LG10 in 2012, where associations between resistance and markers had been observed in other years. Due to the significant effects that the 2012 drought had on measures of fruit quality (S1 Table) and the anomalous KW and IM results (Fig 4B), data from that year were not used and further analysis was based on the combined 2011, 2013 and 2014 data.

**Fig 5:**
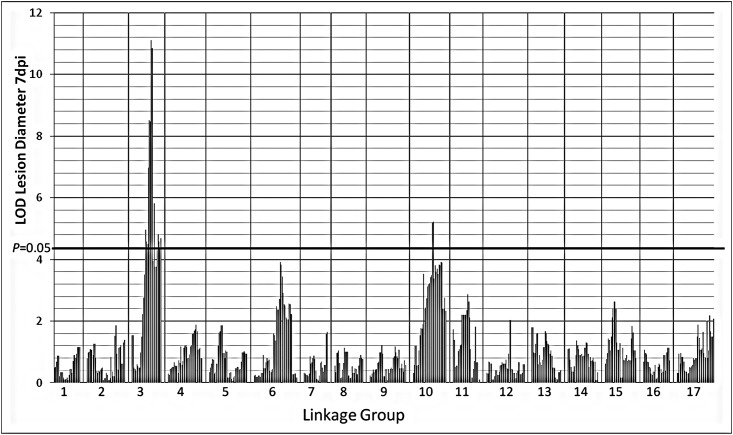
Manhattan plot of interval mapping LOD scores for the association between DNA marker intervals and observed blue mold lesion diameter 7 dpi in the GMAL4593 mapping population identified significant association with DNA markers on LG3 and LG10. The LSmean of lesion diameter 7 dpi was determined from combined 2011, 2013 and 2014 data. The genome wide *P* = 0.05 threshold for the data set was determined to be LOD = 4.4 by a permutation test run (N = 1000) on the genetic linkage map consisting of SSR, SNPlex, HRM, and GBS markers; *P* = 0.05 threshold for LG3 = 2.8, LG6 = 3.0, and LG10 = 3.1.

KW and IM analyses conducted on the combined 2011, 2013, and 2014 data identified significant (*P=* 0.05) associations between resistance to blue mold and markers on LG3 and LG10 (Fig 5). On LG3, significant (*P=* 0.05) LOD scores occurred from 52.7 cM to 74.7 cM, at 83.7 cM, and from 92.9 cM to the end of the LG3 at 98.2 cM (S2 Table). The greatest LOD score (11.1) was observed at 70.2 cM with marker S3_30831583 from PI613981. However, the greatest K value was associated with marker S3_31564599 from PI613981 at 74.7 cM (S2 Table). On LG10 the highest significant (*P=* 0.05) LOD score (5.2) was obtained for two adjacent markers at 73.6 cM (GDsnp00307, PI613981) and 73.8 cM (S10_29121625, ‘Royal Gala’). However, only the ‘Royal Gala’ S10_29121625 marker had a significant K value (GDsnp00307 K = 0.7 and S10_29121625 K = 24.7) (S2 Table).

**Fig 6:**
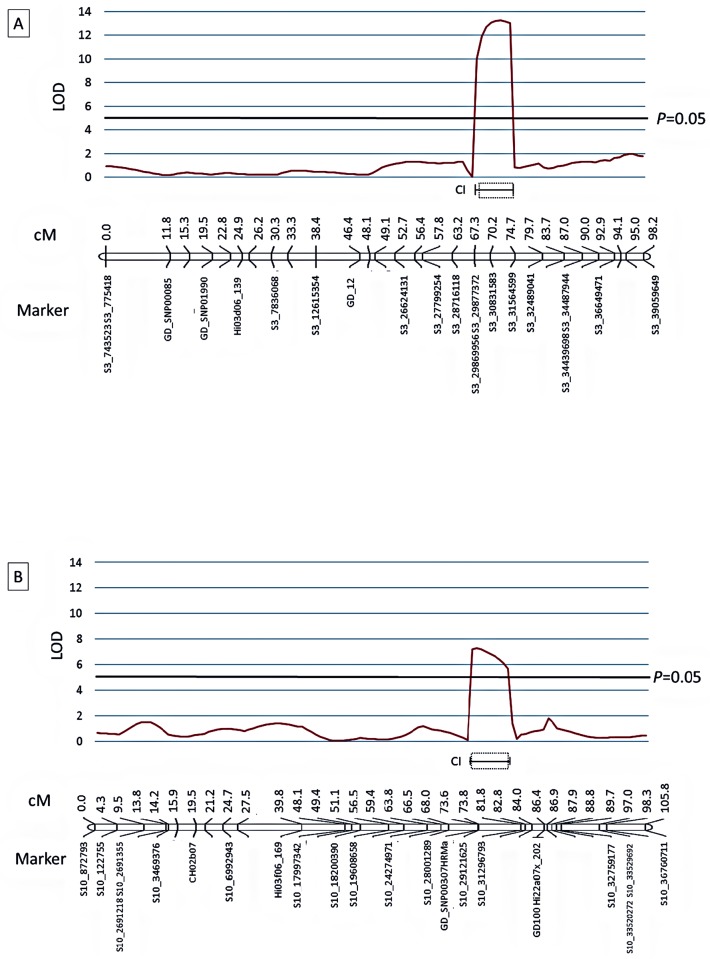
Localization of QTLs for resistance to *P*. *expansum* identified in the GMAL4593 (‘Royal Gala’ X PI613981) mapping population. **A:** qM-*Pe*3.1 on linkage group 3 and **B:** qM-*Pe*10.1 on linkage group 10. The red line trace shows the LOD score determined by multiple QTL modeling using MapQTL6 (Kyazma B.V., Wageningen, The Netherlands). The genome wide *P* = 0.05 threshold for the data set was determined to be LOD = 4.4. The genetic linkage maps are in centiMorgans (cM) and were calculated using JoinMap 4.1 software (Kyazma B.V.). “CI” shows the 90% (solid bracket) and 95% (dashed line box) confidence intervals of the QTLs based upon a LOD support interval method. QTL nomenclature was based on international Rosaceae standards [55].

Based upon the IM results, a multiple QTL model (MQM) analysis [54] was conducted using the combined data from 2011, 2013, and 2014. MQM identified two significant (*P=* 0.05) QTLs for resistance to blue mold on LG3 (qM-*Pe*3.1) and LG10 (qM-*Pe*10.1) (Fig 6). The qM-*Pe*3.1 QTL had a LOD score of 13.3 and accounted for 27.5% of the variation in the data, whereas qM-*Pe*10.1 had a LOD score of 7.4 and accounted for 14% of the variation (S2 Table). qM-*Pe*3.1 contained two GBS markers from the *M*. *sieversii* parent PI613981: S3_29877372 at 67.3 cM and S3_30831583 at 70.2 cM (MQM cofactor). The KW analyses clearly indicated that resistance was being contributed by the PI613981 parent as its markers in the region had highly significant K values (*P=* 0.0001), whereas the ‘Royal Gala’ markers had no significant (*P* = 0.05) association with resistance (S2 Table). The S3_30831583 marker appeared to be the main contributor to LSmean blue mold lesion diameters 7 dpi based upon analysis of parental and recombinant marker haplotypes (S2 Fig), suggesting that the gene(s) in the downstream region of qM-*Pe*3.1 were the main contributors to resistance. This was also supported by both KW and IM analyses, which resulted in highly significant K values and LOD scores for marker S3_31564599 just beyond the MQM downstream stream boundary of qM-*Pe*3.1 (S2 Table).

qM-*Pe*10.1 contained one HRM marker (GDsnp00307HRM, LOD = 7.3) from PI613981 at 73.6 cM and one GBS marker (S10_29121625, LOD = 7.4) from ‘Royal Gala’ at 73.8 cM. However, the KW test was not significant (*P* = 0.05) for the PI613981 GDsnp00307HRM marker and highly significant (*P*<0.0001) for S10_29121625, indicating ‘Royal Gala’ was the primary contributor to qM-*Pe*10.1 resistance (S2 Table). The alleles of GDsnp00307HRM and S10_29121625, however, appeared to act additively in that the combined effect of the positive resistance alleles (GDsnp00307 *np*/ S10_29121625 *ll*) resulted in the lowest LSmean lesion diameter (5.1 mm). In contrast, the combined effect of the negative resistance alleles (GDsnp00307 *nn*/ S10_29121625 *lm*) resulted in the highest (10.1 mm), and positive-negative resistance allele combinations resulted in intermediate LSmean lesion diameters (6.6 mm and 9.1 mm) (S3 Fig).

## Discussion

### Comparison of genetic markers developed from *M*. *× domestica* cultivars and GBS

*M*. *sieversii* is proving to be a valuable resource for the discovery of trait characters not currently found in domesticated apple, as well as for the discovery of new alleles for traits of current importance in breeding programs [56–58]. The genetic diversity that makes *M*. *sieversii* such a valuable resource for apple breeding can also present challenges for its genetic analysis. In the present study, a genetic linkage map composed largely of *M*. × *domestica* SNP markers (65%) was unable to detect a major QTL for resistance to *P*. *expansum* on LG3. Whereas 63% of the *M*. × *domestica* SNPlex markers evaluated were informative for the genetic analysis of ‘Royal Gala’, an offspring of ‘Golden Delicious’, only 22% were informative for *M*. *sieversii* PI613981 (Table 3). Micheletti *et al*. [40] also found that ~88% of 260 *M*. × *domestica* SNPlex markers were informative among *M*. × *domestica* accessions, yet only ~27% of those markers were transferable to *M*. *sieversii* (total of 10 accessions). In contrast, Kumar *et al*. [41] found 86% of a set of 3,521 high quality SNPs from the International RosBREED SNP Consortium apple 8K SNP array [39] were polymorphic in a limited collection of *M*. *sieversii* (16 accessions). The GMAL4593 population in the present study was not genotyped with the International RosBREED SNP Consortium apple 8K SNP array.

In contrast to the *M*. × *domestica* SNP markers, SSR markers previously developed for the genetic analysis of *M*. × *domestica* were equally polymorphic in both ‘Royal Gala’ and PI613981 (Table 3). The HRM markers used in this study were similarly informative for both parents since they were selected based on sequence information from both PI613981 and ‘Golden Delicious’. Genotyping the SSR and HRM markers in the population was, however, labor intensive and primers designed for their detection were generally less than 50% effective, thereby limiting their number in the linkage map. The detection of qM-*Pe*10.1 on LG10 of ‘Royal Gala’ and PI613981 using the SSR/SNPlex/HRM genetic linkage map indicates that markers developed from *M*. × *domestica* did provide adequate coverage for genetic analysis of the ‘Royal Gala’ genome and many portions of the PI613981 genome.

qM-*Pe*3.1 was not detected by the SSR/SNPlex/HRM linkage map due to the map’s poor coverage of the lower portion of LG3 of PI613981. In the ‘Royal Gala’ SSR/SNPlex/HRM linkage map developed in the present study, a total of 16 markers were identified on LG3 covering ~85 cM, whereas only four markers covering ~40 cM were identified in the PI613981 map. Similarly, in an EST contig-based SSR linkage map of a ‘Royal Gala’ X *M*. *sieversii* PI613988 population, Wang *et al*. [58] identified 11 markers that covered ~94 cM of LG3 in the ‘Royal Gala’ map in comparison to five markers covering only ~20 cM in the PI613988 map. A low number of markers was also observed on *M*. *sieversii* LG14 in both the present study (Fig 3A) and Wang *et al*. [58].

There are several possible reasons why the addition of GBS markers to the GMAL4593 genetic linkage map facilitated the identification of qM-*Pe*3.1, including 1) the ability of GBS markers to be more genetic diverse or pedigree-neutral in genotyping exotic genomes due to simultaneous SNP discovery and genotyping, 2) the improved map coverage resulting from the greater number of markers, and 3) the potentially improved map quality resulting from replacement of SNPlex markers having incomplete genotype information (run on only 96 individuals) with GBS markers with more complete data. The GBS protocol resulted in over 1,400 informative markers for the population, which facilitated filling gaps in both the ‘Royal Gala’ and PI613981 maps (Table 4). Although the linkage maps of both parents benefited from the addition of GBS markers, GBS markers seemed to have had a greater impact on the coverage of the PI613981 genome based upon the number of markers per LG (Fig 3). Because GBS markers are generated by direct reads of DNA sequence, they are not dependent upon polymorphisms previously identified in other germplasm, and therefore not dependent upon shared pedigree for SNP detection [44].

It was somewhat surprising that a significantly greater number of GBS markers were informative for ‘Royal Gala’ than PI613981 (Table 4). Because the GBS sequences were not assembled independently but rather aligned to the ‘Golden Delicious’ genome sequence as a reference for SNP identification, only PI613981 genomic regions in common with ‘Golden Delicious’ could be assembled and genotyped. Therefore, the difference in the number of GBS markers between ‘Royal Gala’ and PI613981 could be due to variation in the size and/or structure of the PI613981 genome in comparison to that of the ‘Golden Delicious’ reference. This bias against non-reference species was also noted in half-sib F_1_ *Vitis* families [59]. Since GBS data was aligned to the ‘Golden Delicious’ reference, the results suggest that pM-*Pe*3.1 is present in a genomic region shared by both *M*. × *domestica* and *M*. *sieversii*, but has an allele which is either absent or present at a very low frequency in *M*. × *domestica*.

### QTLs for resistance to *Penicillium expansum*

qM-*Pe*3.1 is the first QTL described for resistance to *P*. *expansum*, the causal agent of blue mold decay of apples, pears and quinces (all in *Maleae*). QTLs for resistance to necrotrophic pathogens have been characterized in relatively few pathosystems and their causative mechanisms of resistance are an area of current research interest [60–63]. Initial studies of differential gene expression in response to challenge of PI613981 and ‘Royal Gala’ with *P*. *expansum* have been conducted [9] and several distinct differences have been noted in regards to the expression of several pathogenesis-related genes and a more detailed analysis of differentially expressed genes that are located on LG3 of apple is near completion (unpublished data). In apple, as in most crop plants, large effect QTLs for disease resistance are often due to genes involved in initial pathogen detection and the induction of a resistance response, rather than specific downstream resistance mechanisms [64–65]. Furthermore, pathogen detection by plants is usually mediated by either direct or indirect monitoring for the presence of pathogen effectors [66]. LysM and NPL1 effectors secreted by *P*. *expansum* have been identified and apple proteins that potentially interact with the identified effectors have also been identified (unpublished data). Further analysis to determine the role of these effectors in the susceptibility of apple to blue mold is also being conducted.

qM-*Pe*10.1 was consistently identified with both the GMAL4593 SSR-SNPlex-HRM and the SSR-SNPlex-HRM-GBS maps. Several genes and QTLs associated with fruit ripening and firmness have been described on LG10 [50] and it is unclear if the effect of qM-*Pe*10.1 on blue mold resistance is due to the pleiotropic effect of fruit ripening or firmness genes on susceptibility to blue mold or resistance factors independent of fruit ripening. Since ‘Royal Gala’ was the primary contributor to the blue mold resistance effect of qM-*Pe*10.1 (S2 Table), this QTL is not likely to be a major contributor to the blue mold resistance of PI613981. However, partitioning the effect of qM-*Pe*10.1 marker alleles on LSmean of blue mold lesion diameter suggests that both alleles are contributing to resistance with additive effects (S3 Fig).

There were several other genomic regions associated with blue mold resistance in the GMAL4593 population that were inconsistent in their occurrence and of lower magnitude. The year to year variation observed in the detection of specific associations (Fig 4) could have been an effect of both yearly environmental effects and the subset of progeny evaluated in each year due to the biennial bearing of many GMAL4593 progeny. Combining the data obtained in different years had the advantage of greater representation of the entire population. Among the potential lesser effect QTLs, the most credible association with blue mold resistance was on LG6. Significant IM LOD scores were observed in 2014 and in the combined data for all years (Fig 4). Although its IM maximum LOD score (3.86) for combined 2011, 2013 and 2014 data did not reach the genome wide *P* = 0.05 significance threshold (Fig 5), it did exceed the LG6 specific *P* = 0.05 threshold (3.1). This potential QTL for resistance to *P*. *expansum* accounted for ~10% of the experimental variation and the resistance allele was contributed by the ‘Royal Gala’ parent. ‘Royal Gala’ has been observed to exhibit greater blue mold resistance than other *M*. *× domestica* cultivars in some years in the Pacific Northwest [11].

The drought conditions of 2012 clearly affected fruit quality and the detection of QTLs for blue mold resistance, so the 2012 data were not included in the final MQM analysis. Two of the significant associations detected by IM in 2012 on LG2 and LG7 were not evident when the 2012 data was removed from the combined year analysis (Fig 5), suggesting they were drought-specific effects on blue mold resistance. A weak association was observed for the third 2012-specific region on LG11 in the Manhattan plot of IM LOD scores even when the 2012 data was not included in the analysis (Fig 5), suggesting its effect on blue mold resistance might not have been drought-specific. The maximal IM LOD score (2.87) on LG11 for the combined data, however, was not significant as it did not reach either the genome wide *P* = 0.05 threshold (Fig 5) or the LG11-specific threshold (3.1). Nearly identical MQM results were obtained when all four years of data were included in the analysis (data not shown), with QTLs for resistance to *P*. *expansum* identified on LG3 and LG10 in the identical locations and only an ~4% decrease in LOD score and proportion of explained variation at LG3 and an ~15% decrease in these measures at LG10. The small decease in qM-*Pe*3.1 LOD score with inclusion of 2012 data suggests that it is a robust locus for resistance to *P*. *expansum* under diverse environmental conditions.

### Breeding blue mold resistant apples cultivars

Although the fruit of *M*. *sieversii* accessions is generally larger and more palatable than other *Malus* sp., the fruit quality of even the more palatable accessions do not meet current industry standards. Therefore, introgression of qM-*Pe*3.1 into elite breeding lines will be required. Historically, the introgression of disease resistance alleles from exotic germplasm into elite breeding lines of fruit tree crops has been a long term process requiring several decades [67]. The use of DNA information to assist in both seedling and parent selection has greatly improved breeding efficiency in apple [68]. A DNA test for qM-*Pe*3.1 has been developed and is currently being evaluated for its ability to predict blue mold resistance in progeny segregating for qM-*Pe*3.1. Due to the long juvenility of apple and the need to evaluate several fruit for reliable determination of blue mold resistance, the availability of a DNA test to screen for the presence of qM-*Pe*3.1 at the seedling stage will greatly improve efficiency of breeding apple for blue mold resistance.

High-speed breeding technology using early flowering transgenic lines of apple has also improved breeding efficiency by reducing generation time from 4–5 years to 6–18 months [69–70]. Crosses have been made between early flowering ‘Pinova’ T1190 [71] and select progeny of GMAL4593 carrying the resistance allele at qM-*Pe*3.1 to both facilitate rapid validation of DNA tests for qM-*Pe*3.1 and accelerate the introgression of resistance alleles into elite breeding lines aided by marker assisted selection [9]. The availability of high quality apple cultivars with resistance to blue mold will enhance the economic viability of the apple industry by reducing production costs and losses, as well as reduce the human health risks associated with pesticide use and patulin production by *P*. *expansum*.

## Conclusions

GBS markers were useful for QTL analysis of an interspecific *Malus* mapping population (*M*. *× domestica* ‘Royal Gala’ X *M*. *sieversii* PI613981) and provided better coverage of the wild species genome than SNP markers developed from domesticated apple cultivars. A large effect QTL controlling resistance to *P*. *expansum*, qM-*Pe*3.1, was identified on LG3 of *M*. *sieversii* PI613981. qM-*Pe*3.1 is the first major QTL described for postharvest blue mold decay. A lesser effect QTL controlling resistance to *P*. *expansum*, qM-*Pe*10.1, was identified on LG10. While ‘Royal Gala’ was the primary contributor to reduced LSmean lesion diameter by qM-*Pe*10.1, alleles from both ‘Royal Gala’ and PI613981 appeared to contribute to resistance in an additive manner.

## Materials and methods

### Determination of resistance to *P*. *expansum*

#### Apple harvest

Fruit was harvested from the GMAL4593 mapping population located on the grounds of the USDA-ARS, Plant Germplasm Resources Research facility, Geneva, NY, USA. At each harvest date fruit maturity was evaluated by visual examination of fruit ground tissue color and seed coat development, resistance to fruit removal from stem and the presence of fruit drops. Since fruit load varied, a range of 60–200 fruit were harvested from each tree and maintained separately. After harvest, fruit was maintained at 4°C in a walk-in cooler, and transported from Geneva, NY to the USDA-ARS facility in Kearneysville, WV where it was also maintained at 4°C until quality parameters and blue mold resistance was assessed.

#### Fruit quality parameters

At each assessment of blue mold resistance, fruit size, firmness, starch, soluble solids, and titrateable acidity was assessed simultaneously on a subset of harvested apples from each genotype. In some cases, the quality parameters were assessed prior to the assessment of blue mold resistance. This was especially true in the later harvest years after it was determined that maturity (mainly predicted by firmness and starch) had an influence on the level of blue mold resistance. Since the objective of the study was to identify resistance that was not associated with maturity, the majority of assessments of blue mold resistance were made when starch and firmness was low. This often necessitated the use of fruit from multiple harvest dates, or leaving the fruit at room temperature for one week prior to assessing blue mold resistance. Fruit mass was used as a measure of fruit size and was determined by weighing a random sample of five apples harvested from each genotype. Firmness of five apples was measured as pounds per square inch, which was then expressed as Newtons, using a Wagner FT 327 penetrometer (Wagner Instruments, Greenwich, CT, USA) mounted on a tabletop drill press. After firmness was assessed, fruit were cut in half, with half of the fruit used to assess starch content and the other half to obtain juice to assess soluble solids and titratable acidity. Starch content was measured as described by Blanpied and Silsby [72]. Juice obtained from five fruit of each genotype was pooled and used to measure soluble solids (degree Brix) with an Atago PR-101 Digital Refractometer (Atago U.S. A., Bellevue, WA, USA). Titrateable acidity was assayed in the pooled juice of five apples of each genotype using a Hanna Instruments HI84432 Titratable Acidity Mini Titrator and pH Meter (HANNA Instruments, Ann Arbor, MI, USA).

#### Resistance to *P*. *expansum*

A suspension of *P*. *expansum* conidia was obtained by flooding a plate of 5–7 d old, actively sporulating culture of an aggressive strain of *P*. *expansum* (strain PE 100) [73], growing on potato dextrose agar (Becton, Dickinson and Co., Sparks, MD), with water to collect spores. The solution was filtered through cheesecloth to remove hyphal debris and then adjusted to a concentration of 1 × 10^4^ spores mL^-1^ with the aid of a hemocytometer. The resulting spore suspension was used to inoculate apples.

Apples were soaked for one min in sodium hypochlorite 200 mg L^-1^ for surface sterilization, rinsed with water, and allowed to dry before being placed into trays. A total of 11 fruit per genotype were used in each test. Fruit were then stored in a growth chamber set at 20°C for 24 h prior to inoculation. A standardized, 3 mm wide and 3 mm deep single nail wound was made in each fruit using a self-made wounding device, and 20 μL of a 1 × 10^4^ spores mL^-1^ spore suspension of *P*. *expansum* was then pipetted into each wound.

The inoculated apples were placed wound-side up on packing trays inside plastic food trays, and covered with plastic lids. Two genotypes were placed in each plastic food tray. The trays were stored at 20°C under high relative humidity maintained by the placement of wet paper towels inside each tray. The paper towels were rehydrated every day. Disease resistance was assessed by measuring lesion diameters on days five and seven.

### Genotyping

#### SSR markers

DNA was isolated from bark tissue collected from dormant, current season’s shoots using a modified CTAB protocol in which powdered tissue suspended in extraction buffer (2% CTAB, 100 mM Tris-HCl, 1.4 M NaCl, 50 mM EDTA and 7% polyvinylpyrrolidone, mol wt. 40,000) was incubated at 70°C for 90 min prior to chloroform extraction and DNA precipitation [74]. PCR reactions were performed using 5’-end FAM, HEX, or NED labeled markers in 96-well plates. Each 15 μL reaction mixture consisted of 2 μL of template DNA (10 ng uL^-1^), 7.5 μL of 2x Phusion GC Master Mix (New England Biolabs, Ipswich, MA), 2 μL of 4 μM mixture of forward and reverse primers, and 3.5 μL of sterile, nuclease-free water. Thermocycler settings were set according to manufacturer instructions and consisted of an initial denaturation step at 98.0°C for 30 s, followed by 40 PCR cycles of 10 s at 98.0°C for (denaturation), 15 s at primer melting temperature (annealing), and a 15 s extension at 72.0°C, followed by a final single 72°C extension and a 4°C hold. Diluted PCR products were run on an ABI 3730 DNA analyzer (ThermoFisher Scientific, Waltham, MA) and the genotype analysis was performed with GeneMapper v3.0 software (ThermoFisher Scientific).

#### SNPlex markers

SNPlex was carried out at Foundation Edmund Mach, IASMA Research Center, San Michele all’Adige, Italy as previously described using fragmented genomic DNA [46]. DNA for SNPlex markers was extracted using a Qiagen DNeasy Plant Mini Kit (Qiagen, Valencia CA, USA).

#### HRM markers

Analysis of HRM markers was performed on a Roche LightCycler 480 (ThermoFisher Scientific, Waltham, MA) using the Roche High Resolution Melting Master Mix Kit, scaled to 10 μL reaction so that each reaction consisted of 2.5 ng of genomic DNA suspended in sterile nuclease-free water at a concentration of 1 ng μL^-1^, 5 μL of HRM Master Mix, 1 μL of MgCl_2_ for a final reaction concentration of 2.5 mM, 1.1 μL sterile nuclease-free water, 0.2 μL of 10 μM stocks of both the forward and reverse primers. Reactions were performed in a 384-well plate and consisted of an initial PCR step followed by a melting step for fluorescence data acquisition. PCR cycling consisted of a 10 m 95°C denaturation step, followed by 40 PCR cycles of 10 s at 95°C, a 10 s touchdown annealing step ranging from 65°C to 55°C with temperature decreasing at a rate of 0.5°C per cycle, and a 72°C extension for 10 s. Heteroduplex formation was initiated by a subsequent heating step at 95°C for 1 min followed by a cooling step at 40°C for 1 min. Melt curve data were obtained by heating the samples over a temperature range of 70°C to 90°C with an acquisition rate of 25 acquisitions per 1°C. Data were analyzed using the LightCycler 480 Gene Scanning software. Melt curve data for each individual along with that of both parents were used to interpret SNP segregation patterns, which were then appropriately coded for incorporation into downstream JoinMap analyses.

#### GBS markers

DNA from 50 mg leaf tissue was extracted using DNeasy 96 Plant Kits (Qiagen, Valencia CA, USA) from ‘Royal Gala’ (PI651008), PI613981 and population GMAL4593 (N = 187). Library construction using *Ape*KI [44] and Illumina HiSeq 2000 (Illumina Inc., San Diego CA, USA) sequencing were completed at the Institute of Biotechnology, Cornell University, Ithaca, NY, USA. Sequence data were processed using the GBS analysis pipeline in TASSEL 3.0 [75]. Sequence tags were aligned to the *M*. × *domestica* Whole Genome Reference Assembly v.2 (https://www.rosaceae.org/) using BWA software [76] under default parameters. Master tag list was created by trimming reads to 64 bp (not including barcodes), and filtering reads with N’s and tags with less than five counts. HapMap genotypes were filtered for site and individuals with more than 20% missing data and converted to JoinMap (Kyazma B.V., Wageningen, The Netherlands) conventions for pseudo-testcross mapping (<lmxll> and <nnxnp>) using R 3.1.1 [77].

### Genetic analysis

#### Genetic linkage map analysis

JoinMap4.1 software (Kyazma B.V., Wageningen, The Netherlands) was used to calculate all genetic linkage maps using default settings. Typical for genetic linkage mapping in cross pollinated species, genotypic data was split into separate maternal (‘Royal Gala’) and paternal (PI613981) datasets using Joinmap4.1 and separate maps were constructed for each parent [78]. Marker order was initially established using a multipoint maximum likelihood algorithm [79] and final map distances were determined using a regression algorithm [80] with the fixed marker order determined from maximum likelihood analysis. Maternal and paternal maps were then integrated using the average map distance of anchor markers present in both parental maps and interpolating the position of markers segregating in only one parent based upon their relative position between the flanking anchor markers [78]. For GBS markers, anchor markers were identified based upon shared proximity (usually within 2 kb pairs) in the *M*. × *domestica* ‘Golden Delicious’ whole genome sequence version 2.0.

Because a large proportion of the SNPlex markers were run on a subset (96) of the 171 population members, for each of the parents two separate genetic linkage maps were developed based on 1) markers run on the entire population (map based on 171 individuals) and 2) all markers using the 96 individuals selected for SNPlex analysis. The parental genetic maps based on 171 and 96 individuals were then integrated for each parent as described above.

The maps containing GBS markers were developed using only markers run on 169 population members (SNPlex markers run on 96 individuals were deleted). To facilitate QTL analysis, genetic linkage maps were developed with markers selected at ca. 5–10 cM intervals, favoring the retention of SSR, GD_SNP and HRM markers over GBS markers.

#### QTL analysis

KW [53] and IM [49] were conducted on the GMAL4593 population in order to identify markers and genomic regions associated with resistance to blue mold. The LSmean of blue mold lesion diameter 7 dpi obtained in each individual year and the combined data over all four years (all years) were used to conduct both analyses. MapQTL 6 software (Kyazma B.V., Wageningen, The Netherlands) was used to conduct KW, IM and MQM. IM and MQM were implemented by a regression approach [54,81]. Marker cofactors used in MQM models were selected using a forward approach where markers with the highest significant LOD score on a specific LG in IM were initially selected and then cofactor selection was similarly repeated in subsequent MQM analysis until the model stabilized. For initial IM analysis, significant LOD value thresholds were estimated based upon previous large-scale simulations [52]. For final IM and MQM analysis, trait dataset and map specific LOD value thresholds (P = 0.05) were empirically established from a set of 1,000 interval mapping iterations run under the null hypothesis of no QTLs (Permutations Test function).

## Supporting information

S1 Fig Images of genetic linkage maps for 17 linkage groups of GMAL4593 population. Images (.pdf) illustrate maps for ‘Royal Gala’ (maternal parent, left), PI613981 (paternal parent, right) and combined GMAL4593 mapping population (center). Anchor markers used to combine parental maps and connected with lines. The genetic linkage maps were calculated using JoinMap4.1 software (Kyazma B.V., Wageningen, The Netherlands).
(PDF)

S2 Fig *M*. *sieversii* PI613981 marker S3_30831583 of LG3 qM-*Pe*3.1 QTL has major effect on the LSmean of blue mold lesion diameter 7 dpi. The parental haplotypes of *M*. *sieversii* PI613981 markers S3_29877372 and S3_30831583 were presumed to be *nn-np* and *np-nn*, respectively, based upon the number of individuals with each haplotypes. Both the *np-nn* parental haplotype and the *nn-nn* recombinant haplotype resulted in a 47% reduction in LSmean lesion diameter suggesting that the downstream region of the QTL is the primary contributor to resistance. Bars represent standard error of the mean.
(PDF)

S3 Fig Effect of LG10 qM-*Pe*10.1 marker allele genotypes on the least square mean (LSmean) of blue mold lesion diameter 7 dpi. **A**: The LSmean lesion diameter of PI613981 GDsnp00307HRM (red tones) and ‘Royal Gala’ S10_29121625 (blue tones) allele genotypes indicated that the *ll* allele of S10_29121625 was the primary contributor of resistance to qM-*Pe*10.1. **B**: The allelic combinations of these markers suggests that the alleles of both parents are acting additively on resistance since the combined positive resistance alleles (*np*,*ll*), combined negative alleles (*nn*,*lm*) and combinations of positive and negative alleles (*nn*,*ll* and *np*,*lm*) had the lowest, highest, and intermediate LSmean lesion diameters, respectively. Bars represent the standard error of the LSmean. The arrows indicate source of alleles.
(PDF)

S1 Table Overall population means of fruit quality parameters in each trial year. Means within a column followed by the same letter did not differ (*P* = 0.05) based upon a Tukey multiple comparison adjustment. Relative starch content was determined using a starch-iodine index chart for ‘Golden Delicious’ [72]. “nt” = not tested.
(PDF)

S2 Table QTL analysis scores for Kruskal-Wallis test, interval mapping and multiple QTL modeling on LG3 and LG10. Source: “RG” = marker heterozygous (informative) only in ‘Royal Gala’, “Ms” = informative only in PI613981, and “Both” = informative in both parents. Mapping LOD scores for “All Years” = score calculated based on blue mold LSmean lesion diameter 7 dpi from all four years of study, whereas “No 2012” = blue mold data from drought year (2012) not included in calculation of LOD score. An “X” in “Cofactor” column indicates marker selected as cofactor in multiple QTL model analysis.
(PDF)

S1 Text Genetic linkage map of GMAL4593 population (‘Royal Gala’ × PI613981). Text file defining 17 LGs with sequential list of markers and marker position in centiMorgans.
(TXT)

S2 Text Genotype data of GMAL4593 genetic linkage map. Text file listing markers and their respective genotype in 169 GMAL4593 progeny used to develop to genetic linkage map. Segregation type: (ab × cd) represents four distinct alleles among the two parents; (ef × eg) represents three alleles with a common allele shared between parents (ab × ac); (hk × hk) represents 2 alleles that are heterozygous in both parents (ab × ab); (lm × ll) represents 2 alleles that are heterozygous only in the maternal parent (ab × aa); and (nn × np) represents 2 alleles that are heterozygous only in the paternal parent (aa × ab).
(TXT)
