# Supplementary material for: Genotyping-by-sequencing markers facilitate the identification of quantitative trait loci controlling resistance to Penicillium expansum in Malus sieversii
Source: PLoS One. 2017 Mar 3;12(3):e0172949. doi: 10.1371/journal.pone.0172949 (PMC5336245; doi:10.1371/journal.pone.0172949)
Supplement: S1 Fig — Images (.pdf) illustrate maps for ‘Royal Gala’ (maternal parent, left), PI613981 (paternal parent, right) and combined GMAL4593 mapping population (center). Anchor markers used to combine parental maps and connected with lines. The genetic linkage maps were calculated using JoinMap4.1 software (Kyazma B.V., Wageningen, The Netherlands). (PDF) [file pone.0172949.s001.pdf]

S1 Fig. Images of genetic linkage maps for 17 linkage groups of GMAL4593 population.

Images illustrate maps for ‘Royal Gala’ (maternal parent, left), PI613981 (paternal parent, right) and combined GMAL4593 mapping population (center).

Anchor markers used to combine parental maps and connected with lines.

The genetic linkage maps were calculated using JoinMap4.1 software (Kyazma B.V., Wageningen, The Netherlands).

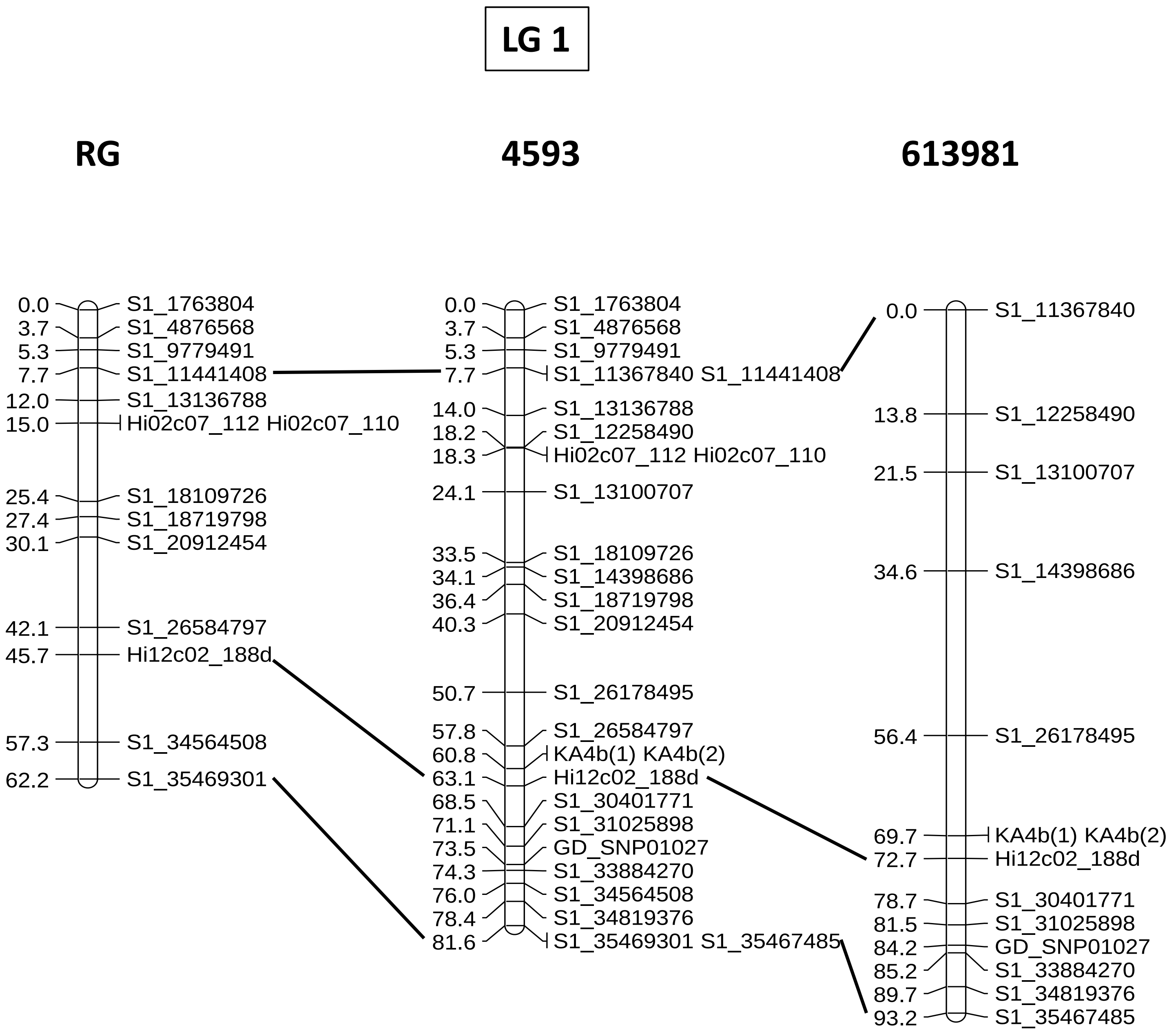
LG 1
RG
4593
613981
0.0 S1_1763804
3.7 S1_4876568
5.3 S1_9779491
7.7 S1_11441408
12.0 S1_13136788
15.0 Hi02c07_112 Hi02c07_110
25.4 S1_18109726
27.4 S1_18719798
30.1 S1_20912454
42.1 S1_26584797
45.7 Hi12c02_188d
57.3 S1_34564508
62.2 S1_35469301
0.0 S1_1763804
3.7 S1_4876568
5.3 S1_9779491
7.7 S1_11367840 S1_11441408
14.0 S1_13136788
18.2 S1_12258490
18.3 Hi02c07_112 Hi02c07_110
24.1 S1_13100707
33.5 S1_18109726
34.1 S1_14398686
36.4 S1_18719798
40.3 S1_20912454
50.7 S1_26178495
57.8 S1_26584797
60.8 KA4b(1) KA4b(2)
63.1 Hi12c02_188d
68.5 S1_30401771
71.1 S1_31025898
73.5 GD_SNP01027
74.3 S1_33884270
76.0 S1_34564508
78.4 S1_34819376
81.6 S1_35469301 S1_35467485
0.0 S1_11367840
13.8 S1_12258490
21.5 S1_13100707
34.6 S1_14398686
56.4 S1_26178495
69.7 KA4b(1) KA4b(2)
72.7 Hi12c02_188d
78.7 S1_30401771
81.5 S1_31025898
84.2 GD_SNP01027
85.2 S1_33884270
89.7 S1_34819376
93.2 S1_35467485

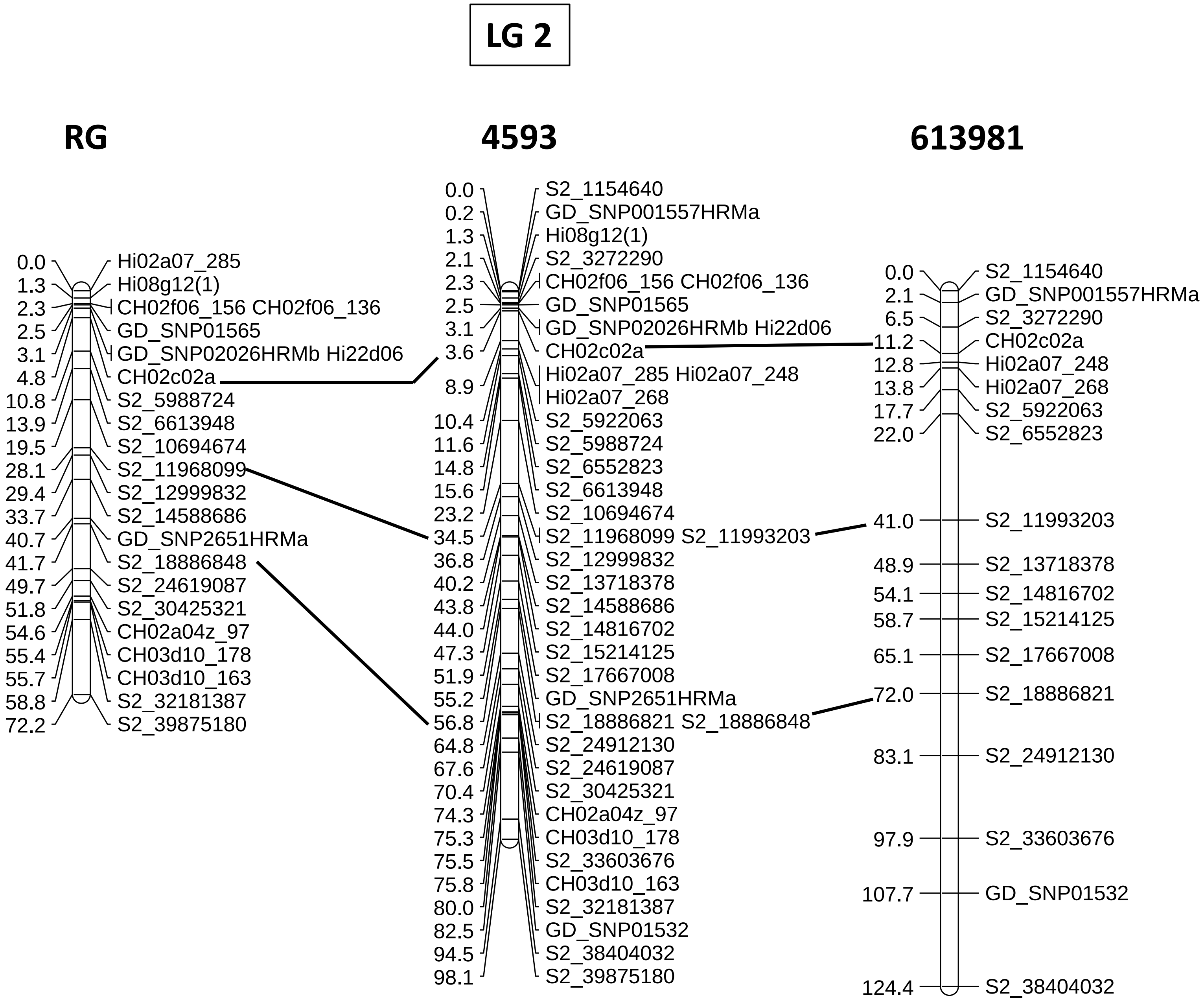
LG 2
RG
4593
613981
0.0 Hi02a07_285
1.3 Hi08g12(1)
2.3 CH02f06_156 CH02f06_136
2.5 GD_SNP01565
3.1 GD_SNP02026HRMb Hi22d06
4.8 CH02c02a
10.8 S2_5988724
13.9 S2_6613948
19.5 S2_10694674
28.1 S2_11968099
29.4 S2_12999832
33.7 S2_14588686
40.7 GD_SNP2651HRMa
41.7 S2_18886848
49.7 S2_24619087
51.8 S2_30425321
54.6 CH02a04z_97
55.4 CH03d10_178
55.7 CH03d10_163
58.8 S2_32181387
72.2 S2_39875180
0.0 S2_1154640
0.2 GD_SNP001557HRMa
1.3 Hi08g12(1)
2.1 S2_3272290
2.3 CH02f06_156 CH02f06_136
2.5 GD_SNP01565
3.1 GD_SNP02026HRMb Hi22d06
3.6 CH02c02a
8.9 Hi02a07_285 Hi02a07_248
Hi02a07_268
10.4 S2_5922063
11.6 S2_5988724
14.8 S2_6552823
15.6 S2_6613948
23.2 S2_10694674
34.5 S2_11968099 S2_11993203
36.8 S2_12999832
40.2 S2_13718378
43.8 S2_14588686
44.0 S2_14816702
47.3 S2_15214125
51.9 S2_17667008
55.2 GD_SNP2651HRMa
56.8 S2_18886821 S2_18886848
64.8 S2_24912130
67.6 S2_24619087
70.4 S2_30425321
74.3 CH02a04z_97
75.3 CH03d10_178
75.5 S2_33603676
75.8 CH03d10_163
80.0 S2_32181387
82.5 GD_SNP01532
94.5 S2_38404032
98.1 S2_39875180
0.0 S2_1154640
2.1 GD_SNP001557HRMa
6.5 S2_3272290
11.2 CH02c02a
12.8 Hi02a07_248
13.8 Hi02a07_268
17.7 S2_5922063
22.0 S2_6552823
41.0 S2_11993203
48.9 S2_13718378
54.1 S2_14816702
58.7 S2_15214125
65.1 S2_17667008
72.0 S2_18886821
83.1 S2_24912130
97.9 S2_33603676
107.7 GD_SNP01532
124.4 S2_38404032

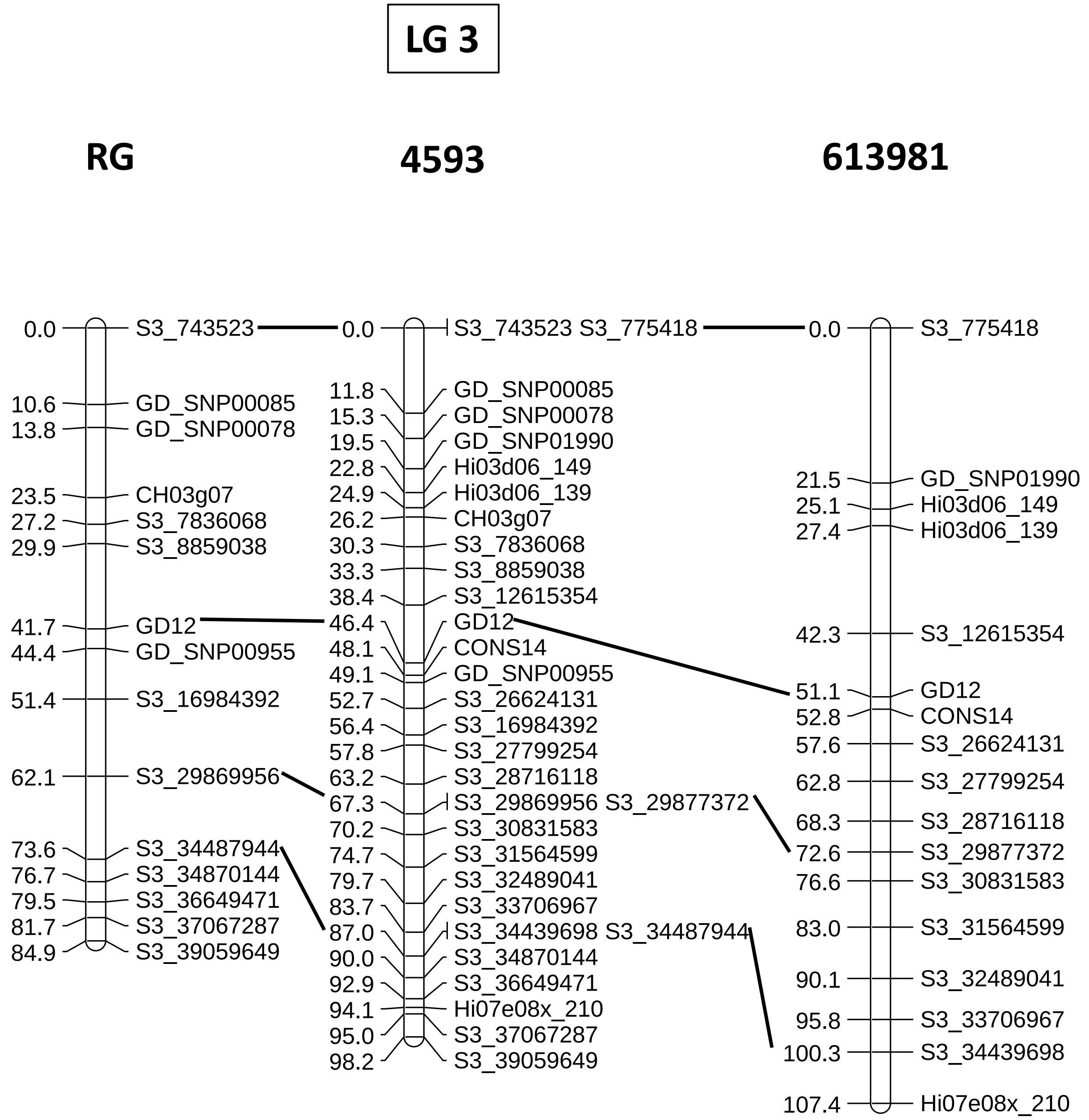
LG 3
RG
4593
613981
0.0 S3_743523
10.6 GD_SNP00085
13.8 GD_SNP00078
23.5 CH03g07
27.2 S3_7836068
29.9 S3_8859038
41.7 GD12
44.4 GD_SNP00955
51.4 S3_16984392
62.1 S3_29869956
73.6 S3_34487944
76.7 S3_34870144
79.5 S3_36649471
81.7 S3_37067287
84.9 S3_39059649
0.0 S3_743523 S3_775418
11.8 GD_SNP00085
15.3 GD_SNP00078
19.5 GD_SNP01990
22.8 Hi03d06_149
24.9 Hi03d06_139
26.2 CH03g07
30.3 S3_7836068
33.3 S3_8859038
38.4 S3_12615354
46.4 GD12
48.1 CONS14
49.1 GD_SNP00955
52.7 S3_26624131
56.4 S3_16984392
57.8 S3_27799254
63.2 S3_28716118
67.3 S3_29869956 S3_29877372
70.2 S3_30831583
74.7 S3_31564599
79.7 S3_32489041
83.7 S3_33706967
87.0 S3_34439698 S3_34487944
90.0 S3_34870144
92.9 S3_36649471
94.1 Hi07e08x_210
95.0 S3_37067287
98.2 S3_39059649
0.0 S3_775418
21.5 GD_SNP01990
25.1 Hi03d06_149
27.4 Hi03d06_139
42.3 S3_12615354
51.1 GD12
52.8 CONS14
57.6 S3_26624131
62.8 S3_27799254
68.3 S3_28716118
72.6 S3_29877372
76.6 S3_30831583
83.0 S3_31564599
90.1 S3_32489041
95.8 S3_33706967
100.3 S3_34439698
107.4 Hi07e08x_210

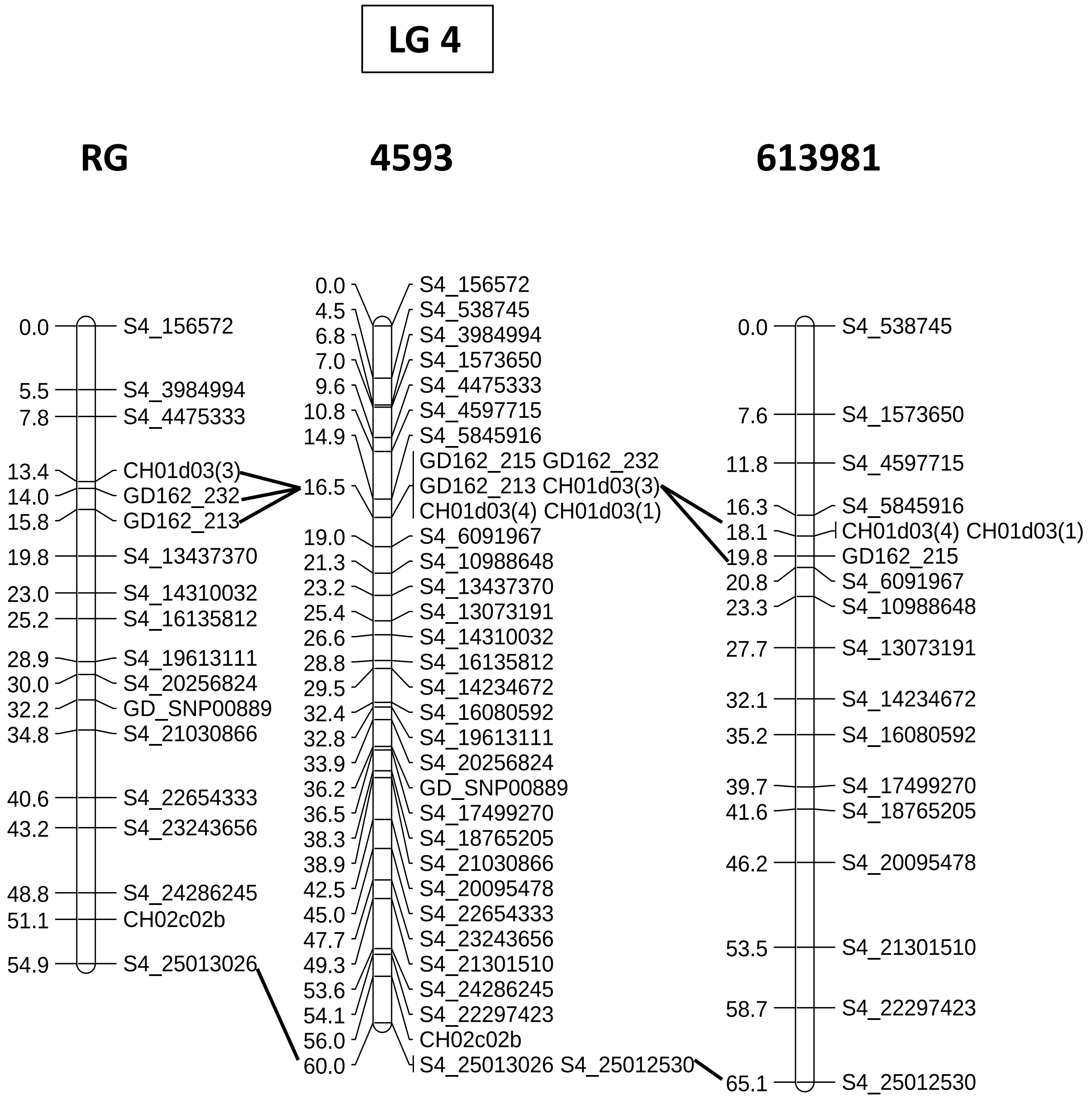
LG 4
RG
4593
613981
0.0 S4_156572
5.5 S4_3984994
7.8 S4_4475333
13.4 CH01d03(3)
14.0 GD162_232
15.8 GD162_213
19.8 S4_13437370
23.0 S4_14310032
25.2 S4_16135812
28.9 S4_19613111
30.0 S4_20256824
32.2 GD_SNP00889
34.8 S4_21030866
40.6 S4_22654333
43.2 S4_23243656
48.8 S4_24286245
51.1 CH02c02b
54.9 S4_25013026
0.0 S4_156572
4.5 S4_538745
6.8 S4_3984994
7.0 S4_1573650
9.6 S4_4475333
10.8 S4_4597715
14.9 S4_5845916
16.5 GD162_215 GD162_232 GD162_213 CH01d03(3) CH01d03(4) CH01d03(1)
19.0 S4_6091967
21.3 S4_10988648
23.2 S4_13437370
25.4 S4_13073191
26.6 S4_14310032
28.8 S4_16135812
29.5 S4_14234672
32.4 S4_16080592
32.8 S4_19613111
33.9 S4_20256824
36.2 GD_SNP00889
36.5 S4_17499270
38.3 S4_18765205
38.9 S4_21030866
42.5 S4_20095478
45.0 S4_22654333
47.7 S4_23243656
49.3 S4_21301510
53.6 S4_24286245
54.1 S4_22297423
56.0 CH02c02b
60.0 S4_25013026 S4_25012530
0.0 S4_538745
7.6 S4_1573650
11.8 S4_4597715
16.3 S4_5845916
18.1 CH01d03(4) CH01d03(1)
19.8 GD162_215
20.8 S4_6091967
23.3 S4_10988648
27.7 S4_13073191
32.1 S4_14234672
35.2 S4_16080592
39.7 S4_17499270
41.6 S4_18765205
46.2 S4_20095478
53.5 S4_21301510
58.7 S4_22297423
65.1 S4_25012530

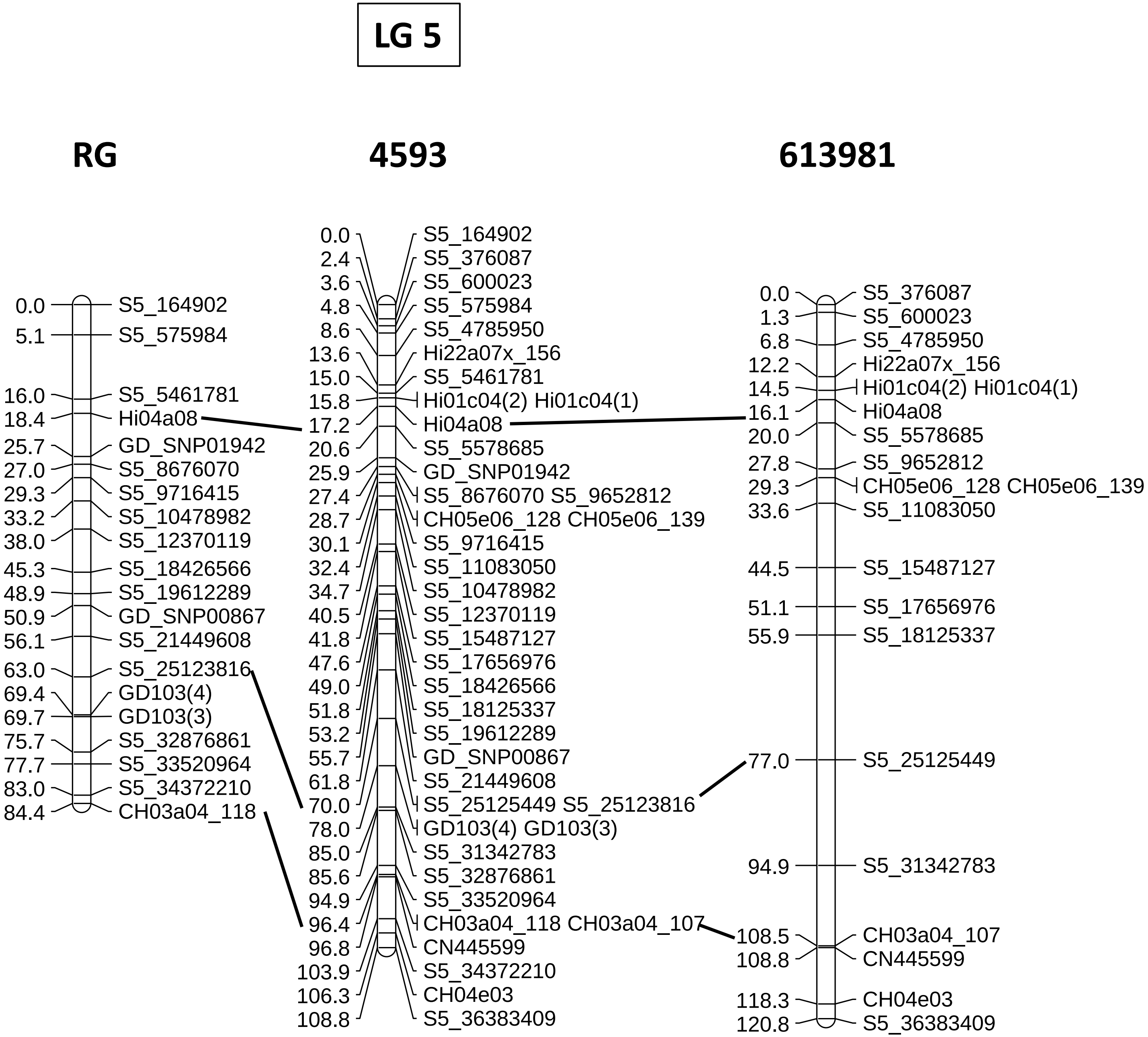

LG 5
RG
0.0 S5_164902
5.1 S5_575984
16.0 S5_5461781
18.4 Hi04a08
25.7 GD_SNP01942
27.0 S5_8676070
29.3 S5_9716415
33.2 S5_10478982
38.0 S5_12370119
45.3 S5_18426566
48.9 S5_19612289
50.9 GD_SNP00867
56.1 S5_21449608
63.0 S5_25123816
69.4 GD103(4)
69.7 GD103(3)
75.7 S5_32876861
77.7 S5_33520964
83.0 S5_34372210
84.4 CH03a04_118
4593
0.0 S5_164902
2.4 S5_376087
3.6 S5_600023
4.8 S5_575984
8.6 S5_4785950
13.6 Hi22a07x_156
15.0 S5_5461781
15.8 Hi01c04(2) Hi01c04(1)
17.2 Hi04a08
20.6 S5_5578685
25.9 GD_SNP01942
27.4 S5_8676070 S5_9652812
28.7 CH05e06_128 CH05e06_139
30.1 S5_9716415
32.4 S5_11083050
34.7 S5_10478982
40.5 S5_12370119
41.8 S5_15487127
47.6 S5_17656976
49.0 S5_18426566
51.8 S5_18125337
53.2 S5_19612289
55.7 GD_SNP00867
61.8 S5_21449608
70.0 S5_25125449 S5_25123816
78.0 GD103(4) GD103(3)
85.0 S5_31342783
85.6 S5_32876861
94.9 S5_33520964
96.4 CH03a04_118 CH03a04_107
96.8 CN445599
103.9 S5_34372210
106.3 CH04e03
108.8 S5_36383409
613981
0.0 S5_376087
1.3 S5_600023
6.8 S5_4785950
12.2 Hi22a07x_156
14.5 Hi01c04(2) Hi01c04(1)
16.1 Hi04a08
20.0 S5_5578685
27.8 S5_9652812
29.3 CH05e06_128 CH05e06_139
33.6 S5_11083050
44.5 S5_15487127
51.1 S5_17656976
55.9 S5_18125337
77.0 S5_25125449
94.9 S5_31342783
108.5 CH03a04_107
108.8 CN445599
118.3 CH04e03
120.8 S5_36383409

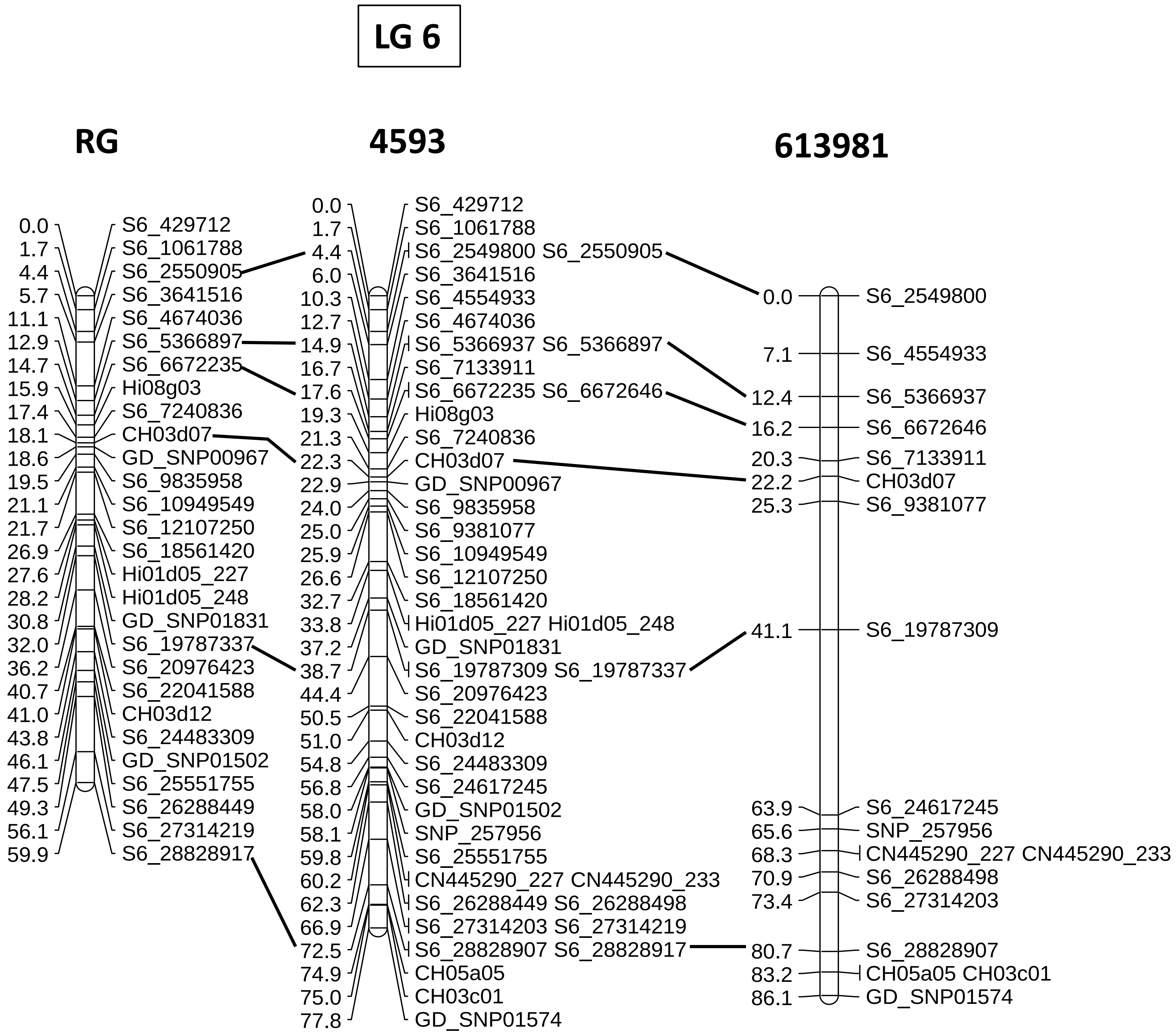
LG 6
RG
4593
613981
0.0 S6_429712
1.7 S6_1061788
4.4 S6_2550905
5.7 S6_3641516
11.1 S6_4674036
12.9 S6_5366897
14.7 S6_6672235
15.9 Hi08g03
17.4 S6_7240836
18.1 CH03d07
18.6 GD_SNP00967
19.5 S6_9835958
21.1 S6_10949549
21.7 S6_12107250
26.9 S6_18561420
27.6 Hi01d05_227
28.2 Hi01d05_248
30.8 GD_SNP01831
32.0 S6_19787337
36.2 S6_20976423
40.7 S6_22041588
41.0 CH03d12
43.8 S6_24483309
46.1 GD_SNP01502
47.5 S6_25551755
49.3 S6_26288449
56.1 S6_27314219
59.9 S6_28828917
0.0 S6_429712
1.7 S6_1061788
4.4 S6_2549800 S6_2550905
6.0 S6_3641516
10.3 S6_4554933
12.7 S6_4674036
14.9 S6_5366937 S6_5366897
16.7 S6_7133911
17.6 S6_6672235 S6_6672646
19.3 Hi08g03
21.3 S6_7240836
22.3 CH03d07
22.9 GD_SNP00967
24.0 S6_9835958
25.0 S6_9381077
25.9 S6_10949549
26.6 S6_12107250
32.7 S6_18561420
33.8 Hi01d05_227 Hi01d05_248
37.2 GD_SNP01831
38.7 S6_19787309 S6_19787337
44.4 S6_20976423
50.5 S6_22041588
51.0 CH03d12
54.8 S6_24483309
56.8 S6_24617245
58.0 GD_SNP01502
58.1 SNP_257956
59.8 S6_25551755
60.2 CN445290_227 CN445290_233
62.3 S6_26288449 S6_26288498
66.9 S6_27314203 S6_27314219
72.5 S6_28828907 S6_28828917
74.9 CH05a05
75.0 CH03c01
77.8 GD_SNP01574
0.0 S6_2549800
7.1 S6_4554933
12.4 S6_5366937
16.2 S6_6672646
20.3 S6_7133911
22.2 CH03d07
25.3 S6_9381077
41.1 S6_19787309
63.9 S6_24617245
65.6 SNP_257956
68.3 CN445290_227 CN445290_233
70.9 S6_26288498
73.4 S6_27314203
80.7 S6_28828907
83.2 CH05a05 CH03c01
86.1 GD_SNP01574

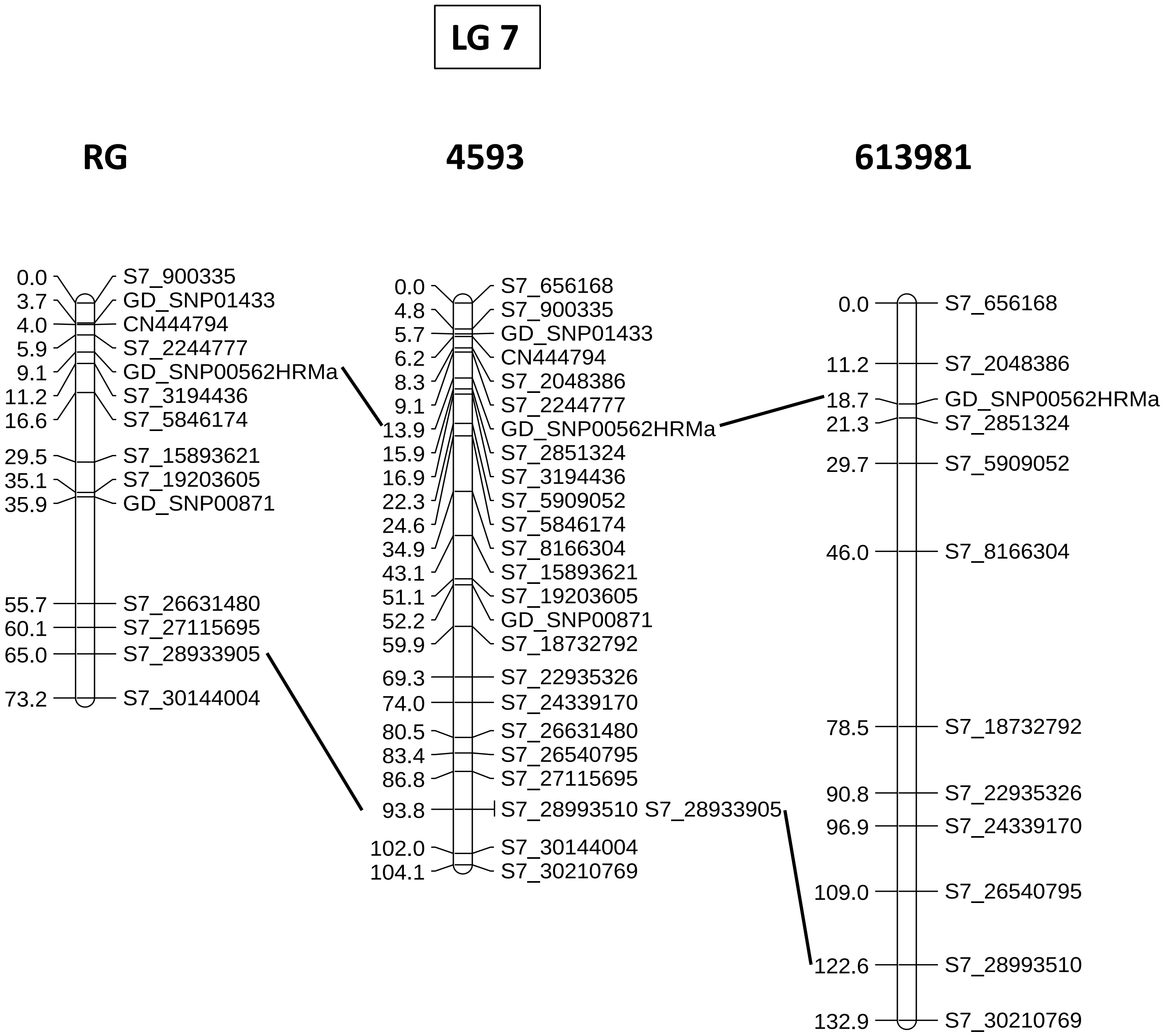
LG 7
RG
4593
613981
0.0 S7_900335
3.7 GD_SNP01433
4.0 CN444794
5.9 S7_2244777
9.1 GD_SNP00562HRMa
11.2 S7_3194436
16.6 S7_5846174
29.5 S7_15893621
35.1 S7_19203605
35.9 GD_SNP00871
55.7 S7_26631480
60.1 S7_27115695
65.0 S7_28933905
73.2 S7_30144004
0.0 S7_656168
4.8 S7_900335
5.7 GD_SNP01433
6.2 CN444794
8.3 S7_2048386
9.1 S7_2244777
13.9 GD_SNP00562HRMa
15.9 S7_2851324
16.9 S7_3194436
22.3 S7_5909052
24.6 S7_5846174
34.9 S7_8166304
43.1 S7_15893621
51.1 S7_19203605
52.2 GD_SNP00871
59.9 S7_18732792
69.3 S7_22935326
74.0 S7_24339170
80.5 S7_26631480
83.4 S7_26540795
86.8 S7_27115695
93.8 S7_28993510 S7_28933905
102.0 S7_30144004
104.1 S7_30210769
0.0 S7_656168
11.2 S7_2048386
18.7 GD_SNP00562HRMa
21.3 S7_2851324
29.7 S7_5909052
46.0 S7_8166304
78.5 S7_18732792
90.8 S7_22935326
96.9 S7_24339170
109.0 S7_26540795
122.6 S7_28993510
132.9 S7_30210769

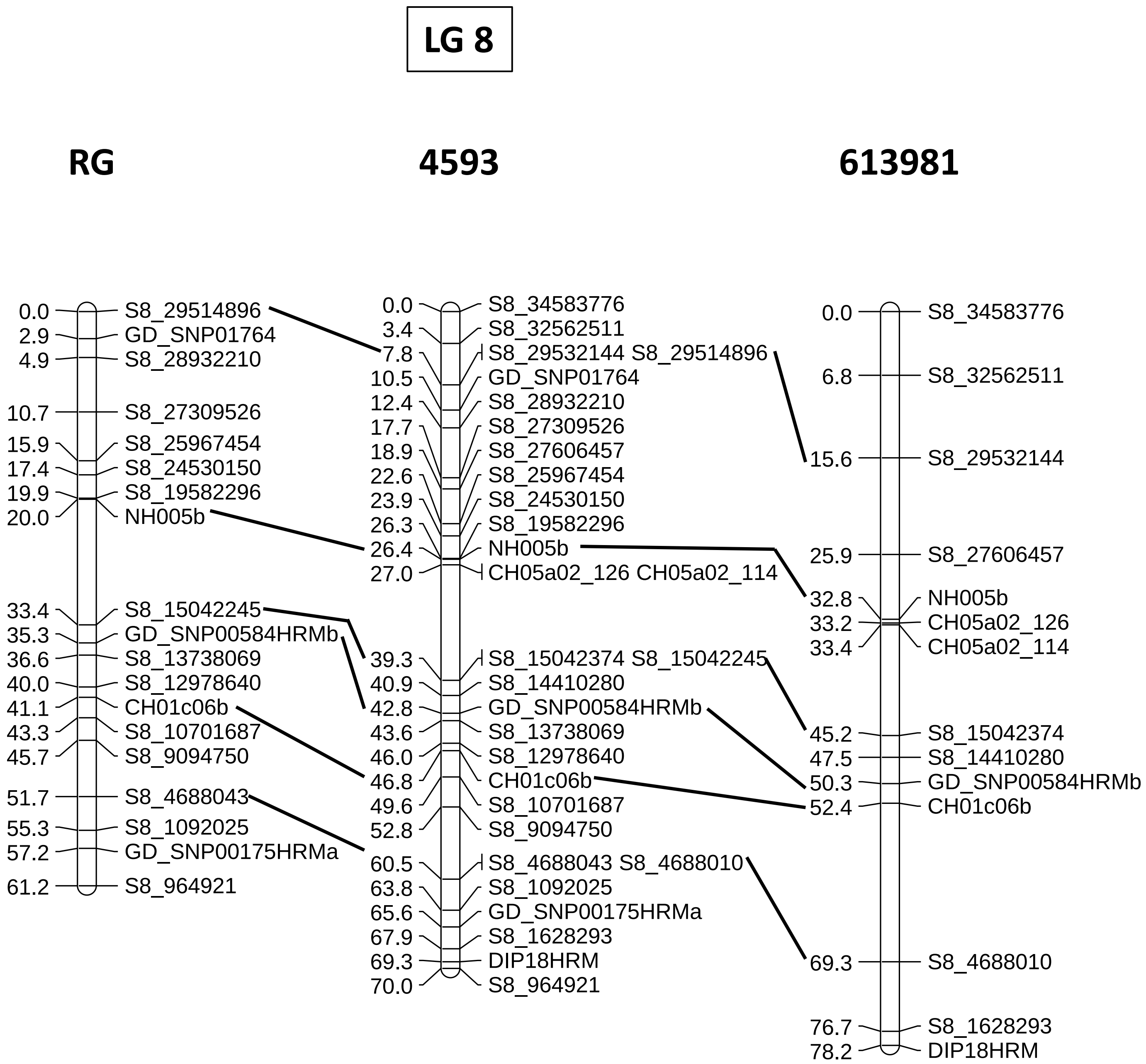
LG 8
RG
4593
613981
0.0 S8_29514896
2.9 GD_SNP01764
4.9 S8_28932210
10.7 S8_27309526
15.9 S8_25967454
17.4 S8_24530150
19.9 S8_19582296
20.0 NH005b
33.4 S8_15042245
35.3 GD_SNP00584HRMb
36.6 S8_13738069
40.0 S8_12978640
41.1 CH01c06b
43.3 S8_10701687
45.7 S8_9094750
51.7 S8_4688043
55.3 S8_1092025
57.2 GD_SNP00175HRMa
61.2 S8_964921
0.0 S8_34583776
3.4 S8_32562511
7.8 S8_29532144 S8_29514896
10.5 GD_SNP01764
12.4 S8_28932210
17.7 S8_27309526
18.9 S8_27606457
22.6 S8_25967454
23.9 S8_24530150
26.3 S8_19582296
26.4 NH005b
27.0 CH05a02_126 CH05a02_114
39.3 S8_15042374 S8_15042245
40.9 S8_14410280
42.8 GD_SNP00584HRMb
43.6 S8_13738069
46.0 S8_12978640
46.8 CH01c06b
49.6 S8_10701687
52.8 S8_9094750
60.5 S8_4688043 S8_4688010
63.8 S8_1092025
65.6 GD_SNP00175HRMa
67.9 S8_1628293
69.3 DIP18HRM
70.0 S8_964921
0.0 S8_34583776
6.8 S8_32562511
15.6 S8_29532144
25.9 S8_27606457
32.8 NH005b
33.2 CH05a02_126
33.4 CH05a02_114
45.2 S8_15042374
47.5 S8_14410280
50.3 GD_SNP00584HRMb
52.4 CH01c06b
69.3 S8_4688010
76.7 S8_1628293
78.2 DIP18HRM

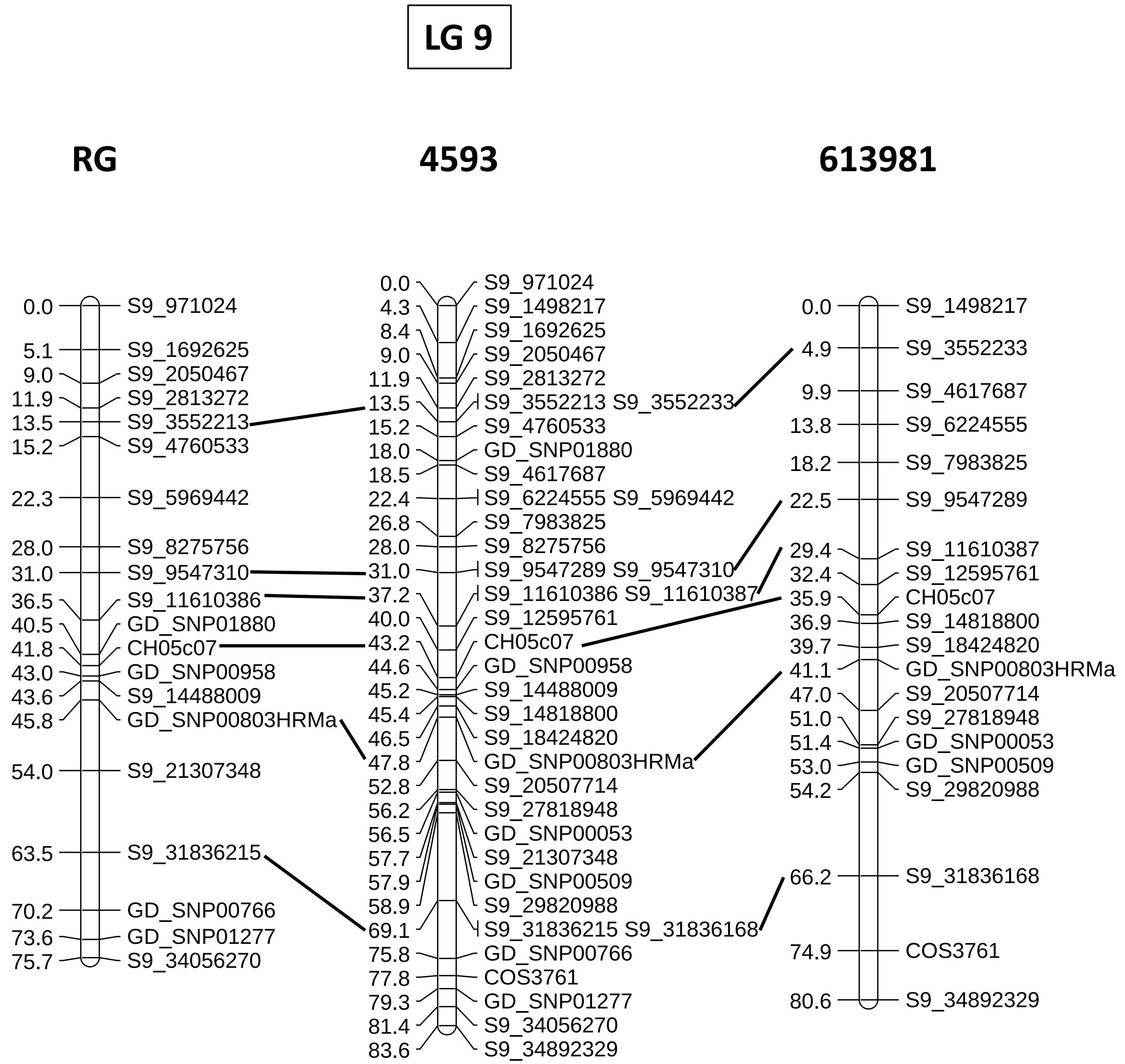

LG 9
RG
4593
613981
0.0 S9_971024
5.1 S9_1692625
9.0 S9_2050467
11.9 S9_2813272
13.5 S9_3552213
15.2 S9_4760533
22.3 S9_5969442
28.0 S9_8275756
31.0 S9_9547310
36.5 S9_11610386
40.5 GD_SNP01880
41.8 CH05c07
43.0 GD_SNP00958
43.6 S9_14488009
45.8 GD_SNP00803HRMa
54.0 S9_21307348
63.5 S9_31836215
70.2 GD_SNP00766
73.6 GD_SNP01277
75.7 S9_34056270
0.0 S9_971024
4.3 S9_1498217
8.4 S9_1692625
9.0 S9_2050467
11.9 S9_2813272
13.5 S9_3552213 S9_3552233
15.2 S9_4760533
18.0 GD_SNP01880
18.5 S9_4617687
22.4 S9_6224555 S9_5969442
26.8 S9_7983825
28.0 S9_8275756
31.0 S9_9547289 S9_9547310
37.2 S9_11610386 S9_11610387
40.0 S9_12595761
43.2 CH05c07
44.6 GD_SNP00958
45.2 S9_14488009
45.4 S9_14818800
46.5 S9_18424820
47.8 GD_SNP00803HRMa
52.8 S9_20507714
56.2 S9_27818948
56.5 GD_SNP00053
57.7 S9_21307348
57.9 GD_SNP00509
58.9 S9_29820988
69.1 S9_31836215 S9_31836168
75.8 GD_SNP00766
77.8 COS3761
79.3 GD_SNP01277
81.4 S9_34056270
83.6 S9_34892329
0.0 S9_1498217
4.9 S9_3552233
9.9 S9_4617687
13.8 S9_6224555
18.2 S9_7983825
22.5 S9_9547289
29.4 S9_11610387
32.4 S9_12595761
35.9 CH05c07
36.9 S9_14818800
39.7 S9_18424820
41.1 GD_SNP00803HRMa
47.0 S9_20507714
51.0 S9_27818948
51.4 GD_SNP00053
53.0 GD_SNP00509
54.2 S9_29820988
66.2 S9_31836168
74.9 COS3761
80.6 S9_34892329

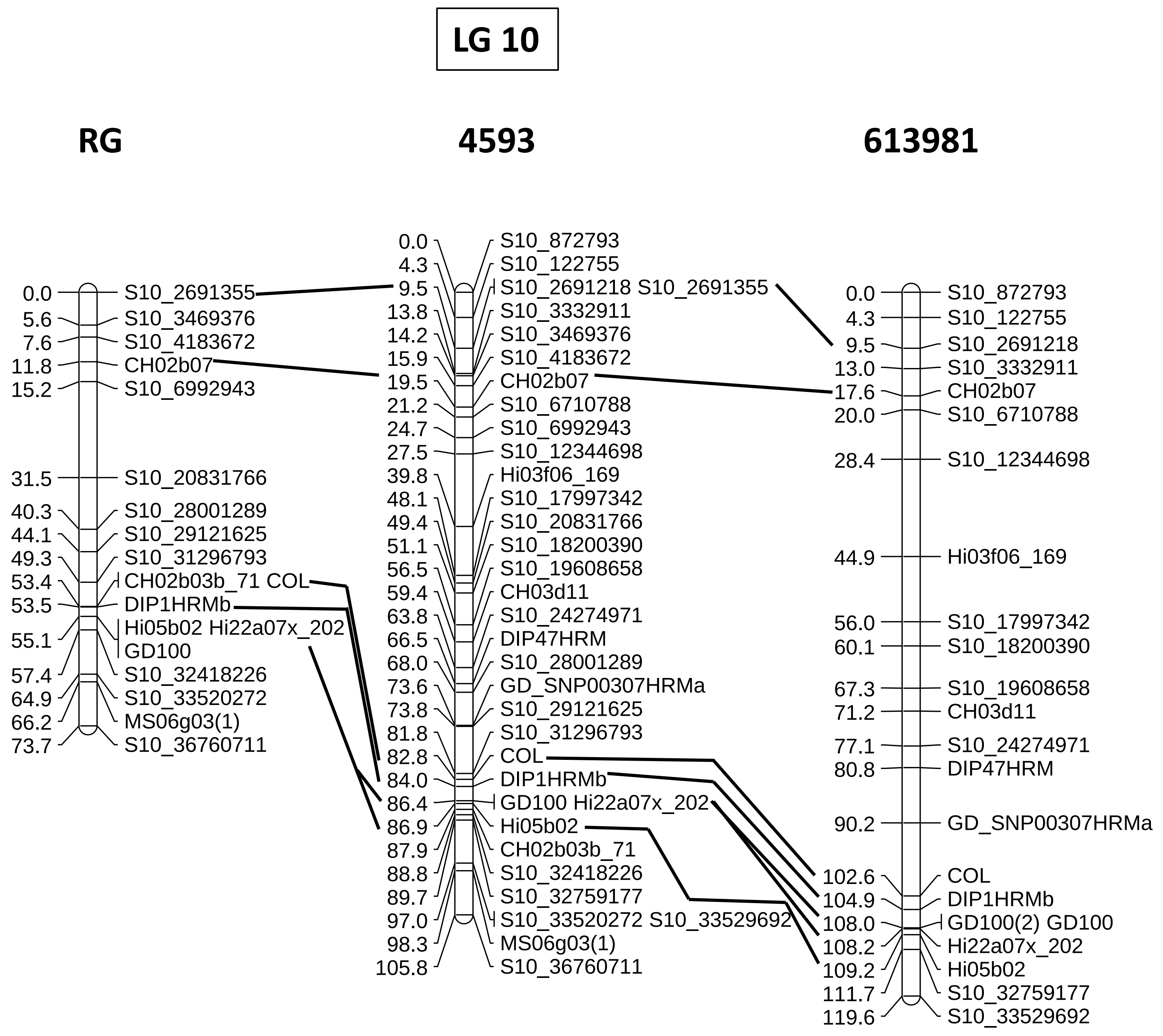
LG 10
RG
0.0 S10_2691355
5.6 S10_3469376
7.6 S10_4183672
11.8 CH02b07
15.2 S10_6992943
31.5 S10_20831766
40.3 S10_28001289
44.1 S10_29121625
49.3 S10_31296793
53.4 CH02b03b_71 COL
53.5 DIP1HRMb
55.1 Hi05b02 Hi22a07x_202 GD100
57.4 S10_32418226
64.9 S10_33520272
66.2 MS06g03(1)
73.7 S10_36760711
4593
0.0 S10_872793
4.3 S10_122755
9.5 S10_2691218 S10_2691355
13.8 S10_3332911
14.2 S10_3469376
15.9 S10_4183672
19.5 CH02b07
21.2 S10_6710788
24.7 S10_6992943
27.5 S10_12344698
39.8 Hi03f06_169
48.1 S10_17997342
49.4 S10_20831766
51.1 S10_18200390
56.5 S10_19608658
59.4 CH03d11
63.8 S10_24274971
66.5 DIP47HRM
68.0 S10_28001289
73.6 GD_SNP00307HRMa
73.8 S10_29121625
81.8 S10_31296793
82.8 COL
84.0 DIP1HRMb
86.4 GD100 Hi22a07x_202
86.9 Hi05b02
87.9 CH02b03b_71
88.8 S10_32418226
89.7 S10_32759177
97.0 S10_33520272 S10_33529692
98.3 MS06g03(1)
105.8 S10_36760711
613981
0.0 S10_872793
4.3 S10_122755
9.5 S10_2691218
13.0 S10_3332911
17.6 CH02b07
20.0 S10_6710788
28.4 S10_12344698
44.9 Hi03f06_169
56.0 S10_17997342
60.1 S10_18200390
67.3 S10_19608658
71.2 CH03d11
77.1 S10_24274971
80.8 DIP47HRM
90.2 GD_SNP00307HRMa
102.6 COL
104.9 DIP1HRMb
108.0 GD100(2) GD100
108.2 Hi22a07x_202
109.2 Hi05b02
111.7 S10_32759177
119.6 S10_33529692

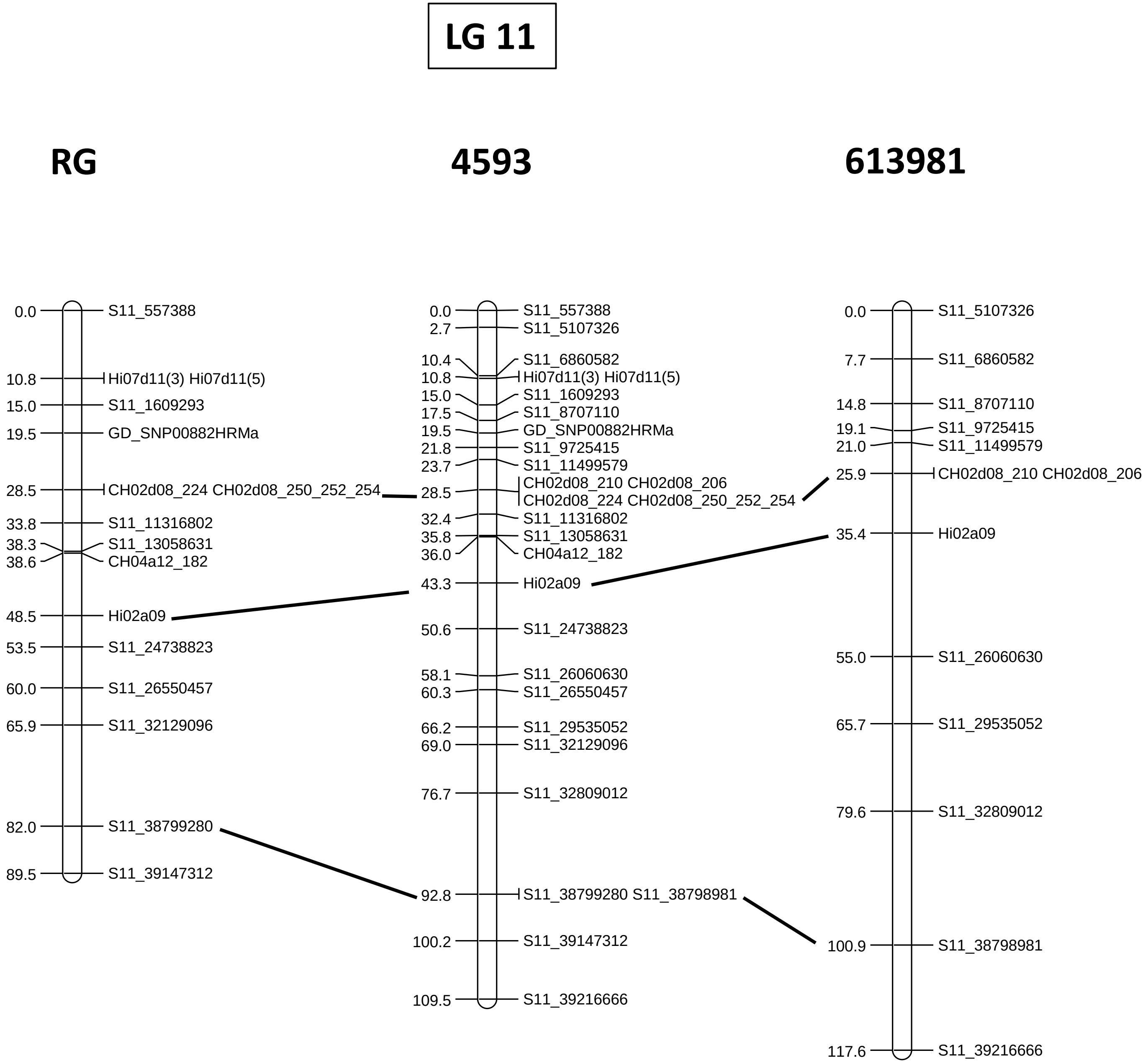

LG 11
RG
4593
613981
0.0 S11_557388
10.8 Hi07d11(3) Hi07d11(5)
15.0 S11_1609293
19.5 GD_SNP00882HRMa
28.5 CH02d08_224 CH02d08_250_252_254
33.8 S11_11316802
38.3 S11_13058631
38.6 CH04a12_182
48.5 Hi02a09
53.5 S11_24738823
60.0 S11_26550457
65.9 S11_32129096
82.0 S11_38799280
89.5 S11_39147312
0.0 S11_557388
2.7 S11_5107326
10.4 S11_6860582
10.8 Hi07d11(3) Hi07d11(5)
15.0 S11_1609293
17.5 S11_8707110
19.5 GD_SNP00882HRMa
21.8 S11_9725415
23.7 S11_11499579
28.5 CH02d08_210 CH02d08_206 CH02d08_224 CH02d08_250_252_254
32.4 S11_11316802
35.8 S11_13058631
36.0 CH04a12_182
43.3 Hi02a09
50.6 S11_24738823
58.1 S11_26060630
60.3 S11_26550457
66.2 S11_29535052
69.0 S11_32129096
76.7 S11_32809012
92.8 S11_38799280 S11_38798981
100.2 S11_39147312
109.5 S11_39216666
0.0 S11_5107326
7.7 S11_6860582
14.8 S11_8707110
19.1 S11_9725415
21.0 S11_11499579
25.9 CH02d08_210 CH02d08_206
35.4 Hi02a09
55.0 S11_26060630
65.7 S11_29535052
79.6 S11_32809012
100.9 S11_38798981
117.6 S11_39216666

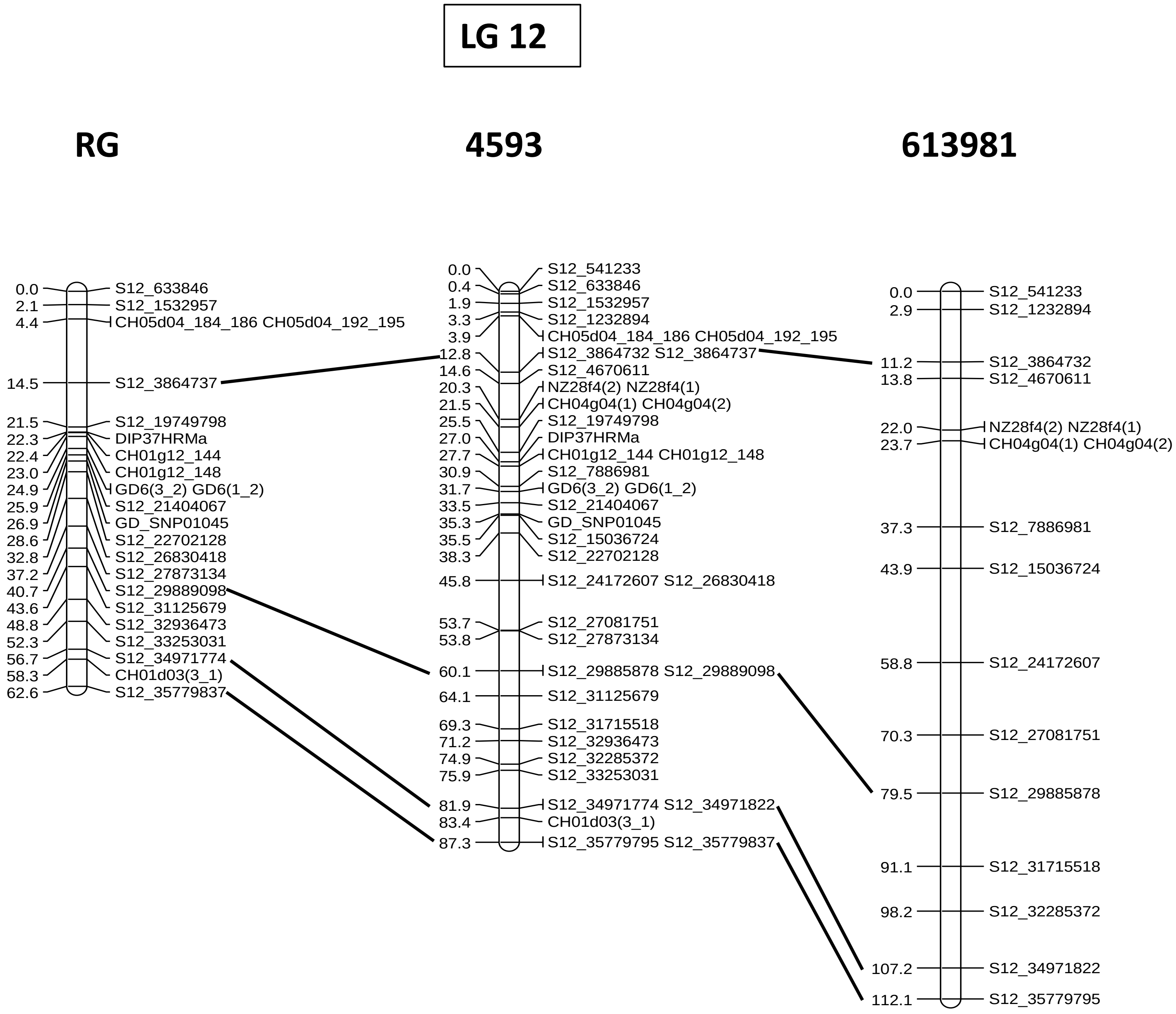

LG 12
RG
4593
613981
0.0 S12_633846
2.1 S12_1532957
4.4 CH05d04_184_186 CH05d04_192_195
14.5 S12_3864737
21.5 S12_19749798
22.3 DIP37HRMa
22.4 CH01g12_144
23.0 CH01g12_148
24.9 GD6(3_2) GD6(1_2)
25.9 S12_21404067
26.9 GD_SNP01045
28.6 S12_22702128
32.8 S12_26830418
37.2 S12_27873134
40.7 S12_29889098
43.6 S12_31125679
48.8 S12_32936473
52.3 S12_33253031
56.7 S12_34971774
58.3 CH01d03(3_1)
62.6 S12_35779837
0.0 S12_541233
0.4 S12_633846
1.9 S12_1532957
3.3 S12_1232894
3.9 CH05d04_184_186 CH05d04_192_195
12.8 S12_3864732 S12_3864737
14.6 S12_4670611
20.3 NZ28f4(2) NZ28f4(1)
21.5 CH04g04(1) CH04g04(2)
25.5 S12_19749798
27.0 DIP37HRMa
27.7 CH01g12_144 CH01g12_148
30.9 S12_7886981
31.7 GD6(3_2) GD6(1_2)
33.5 S12_21404067
35.3 GD_SNP01045
35.5 S12_15036724
38.3 S12_22702128
45.8 S12_24172607 S12_26830418
53.7 S12_27081751
53.8 S12_27873134
60.1 S12_29885878 S12_29889098
64.1 S12_31125679
69.3 S12_31715518
71.2 S12_32936473
74.9 S12_32285372
75.9 S12_33253031
81.9 S12_34971774 S12_34971822
83.4 CH01d03(3_1)
87.3 S12_35779795 S12_35779837
0.0 S12_541233
2.9 S12_1232894
11.2 S12_3864732
13.8 S12_4670611
22.0 NZ28f4(2) NZ28f4(1)
23.7 CH04g04(1) CH04g04(2)
37.3 S12_7886981
43.9 S12_15036724
58.8 S12_24172607
70.3 S12_27081751
79.5 S12_29885878
91.1 S12_31715518
98.2 S12_32285372
107.2 S12_34971822
112.1 S12_35779795

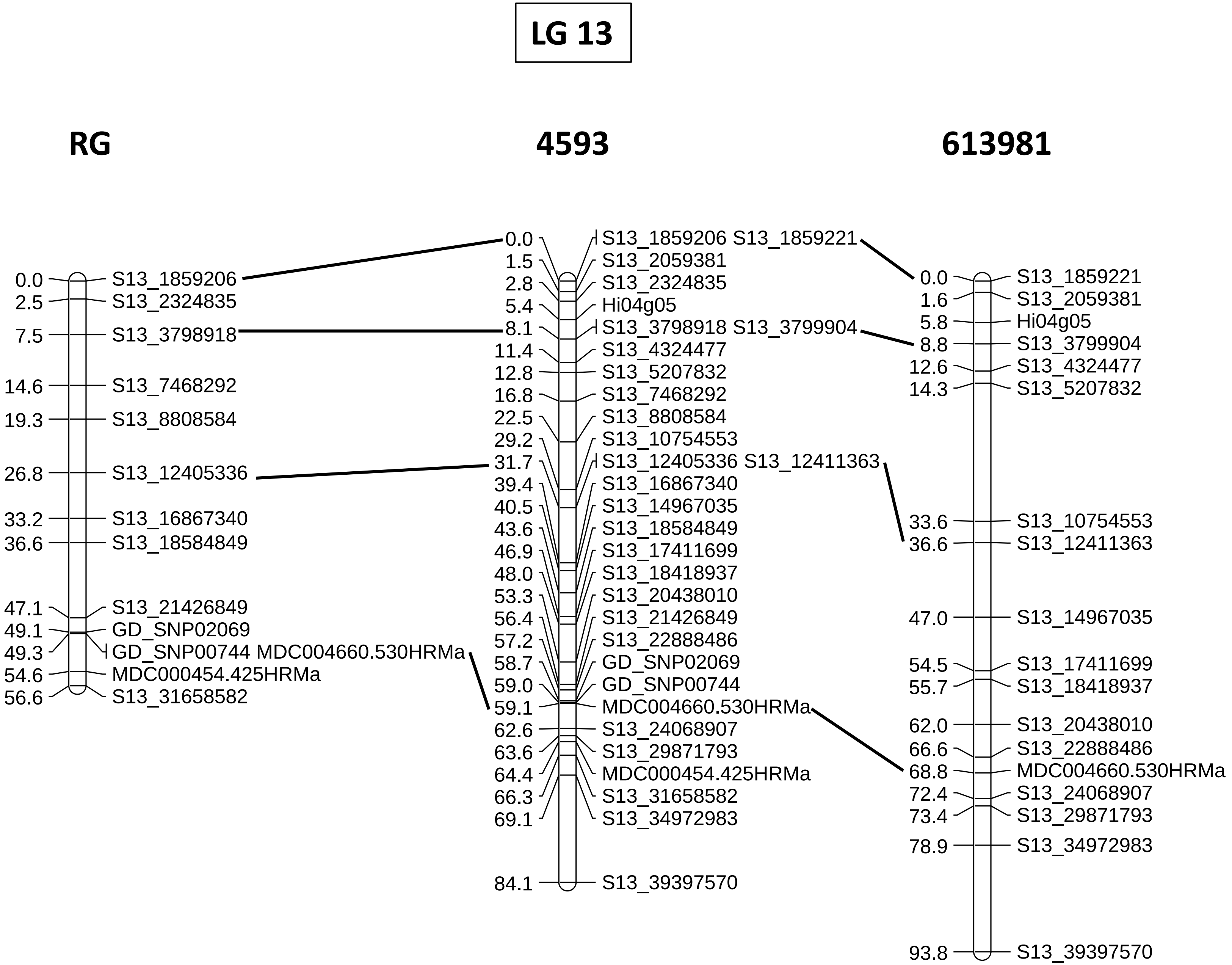

LG 13
RG
4593
613981
0.0 S13_1859206
2.5 S13_2324835
7.5 S13_3798918
14.6 S13_7468292
19.3 S13_8808584
26.8 S13_12405336
33.2 S13_16867340
36.6 S13_18584849
47.1 S13_21426849
49.1 GD_SNP02069
49.3 GD_SNP00744 MDC004660.530HRMa
54.6 MDC000454.425HRMa
56.6 S13_31658582
0.0 S13_1859206 S13_1859221
1.5 S13_2059381
2.8 S13_2324835
5.4 Hi04g05
8.1 S13_3798918 S13_3799904
11.4 S13_4324477
12.8 S13_5207832
16.8 S13_7468292
22.5 S13_8808584
29.2 S13_10754553
31.7 S13_12405336 S13_12411363
39.4 S13_16867340
40.5 S13_14967035
43.6 S13_18584849
46.9 S13_17411699
48.0 S13_18418937
53.3 S13_20438010
56.4 S13_21426849
57.2 S13_22888486
58.7 GD_SNP02069
59.0 GD_SNP00744
59.1 MDC004660.530HRMa
62.6 S13_24068907
63.6 S13_29871793
64.4 MDC000454.425HRMa
66.3 S13_31658582
69.1 S13_34972983
84.1 S13_39397570
0.0 S13_1859221
1.6 S13_2059381
5.8 Hi04g05
8.8 S13_3799904
12.6 S13_4324477
14.3 S13_5207832
33.6 S13_10754553
36.6 S13_12411363
47.0 S13_14967035
54.5 S13_17411699
55.7 S13_18418937
62.0 S13_20438010
66.6 S13_22888486
68.8 MDC004660.530HRMa
72.4 S13_24068907
73.4 S13_29871793
78.9 S13_34972983
93.8 S13_39397570

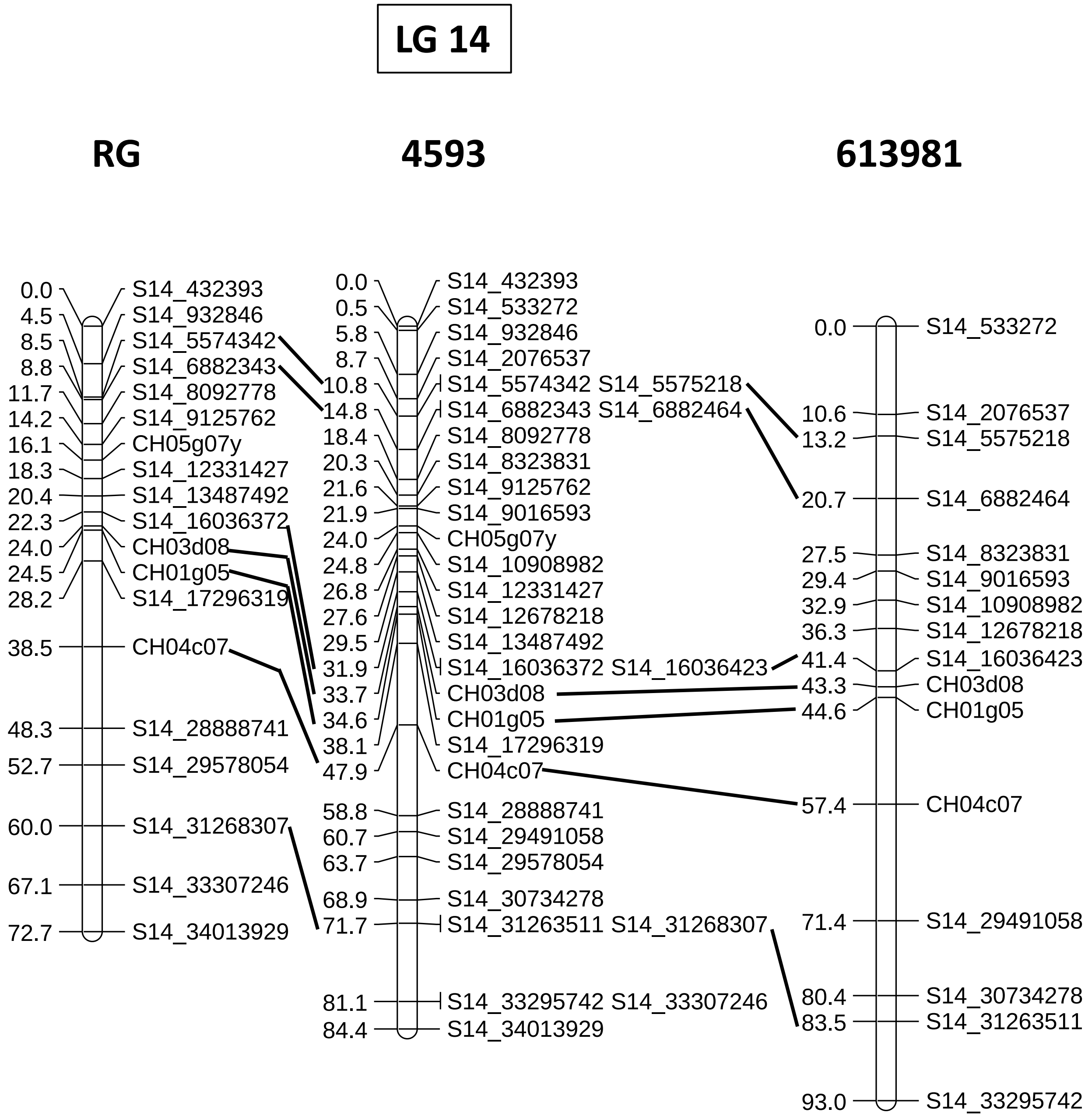
LG 14
RG
4593
613981
0.0 S14_432393
4.5 S14_932846
8.5 S14_5574342
8.8 S14_6882343
11.7 S14_8092778
14.2 S14_9125762
16.1 CH05g07y
18.3 S14_12331427
20.4 S14_13487492
22.3 S14_16036372
24.0 CH03d08
24.5 CH01g05
28.2 S14_17296319
38.5 CH04c07
48.3 S14_28888741
52.7 S14_29578054
60.0 S14_31268307
67.1 S14_33307246
72.7 S14_34013929
0.0 S14_432393
0.5 S14_533272
5.8 S14_932846
8.7 S14_2076537
10.8 S14_5574342 S14_5575218
14.8 S14_6882343 S14_6882464
18.4 S14_8092778
20.3 S14_8323831
21.6 S14_9125762
21.9 S14_9016593
24.0 CH05g07y
24.8 S14_10908982
26.8 S14_12331427
27.6 S14_12678218
29.5 S14_13487492
31.9 S14_16036372 S14_16036423
33.7 CH03d08
34.6 CH01g05
38.1 S14_17296319
47.9 CH04c07
58.8 S14_28888741
60.7 S14_29491058
63.7 S14_29578054
68.9 S14_30734278
71.7 S14_31263511 S14_31268307
81.1 S14_33295742 S14_33307246
84.4 S14_34013929
0.0 S14_533272
10.6 S14_2076537
13.2 S14_5575218
20.7 S14_6882464
27.5 S14_8323831
29.4 S14_9016593
32.9 S14_10908982
36.3 S14_12678218
41.4 S14_16036423
43.3 CH03d08
44.6 CH01g05
57.4 CH04c07
71.4 S14_29491058
80.4 S14_30734278
83.5 S14_31263511
93.0 S14_33295742

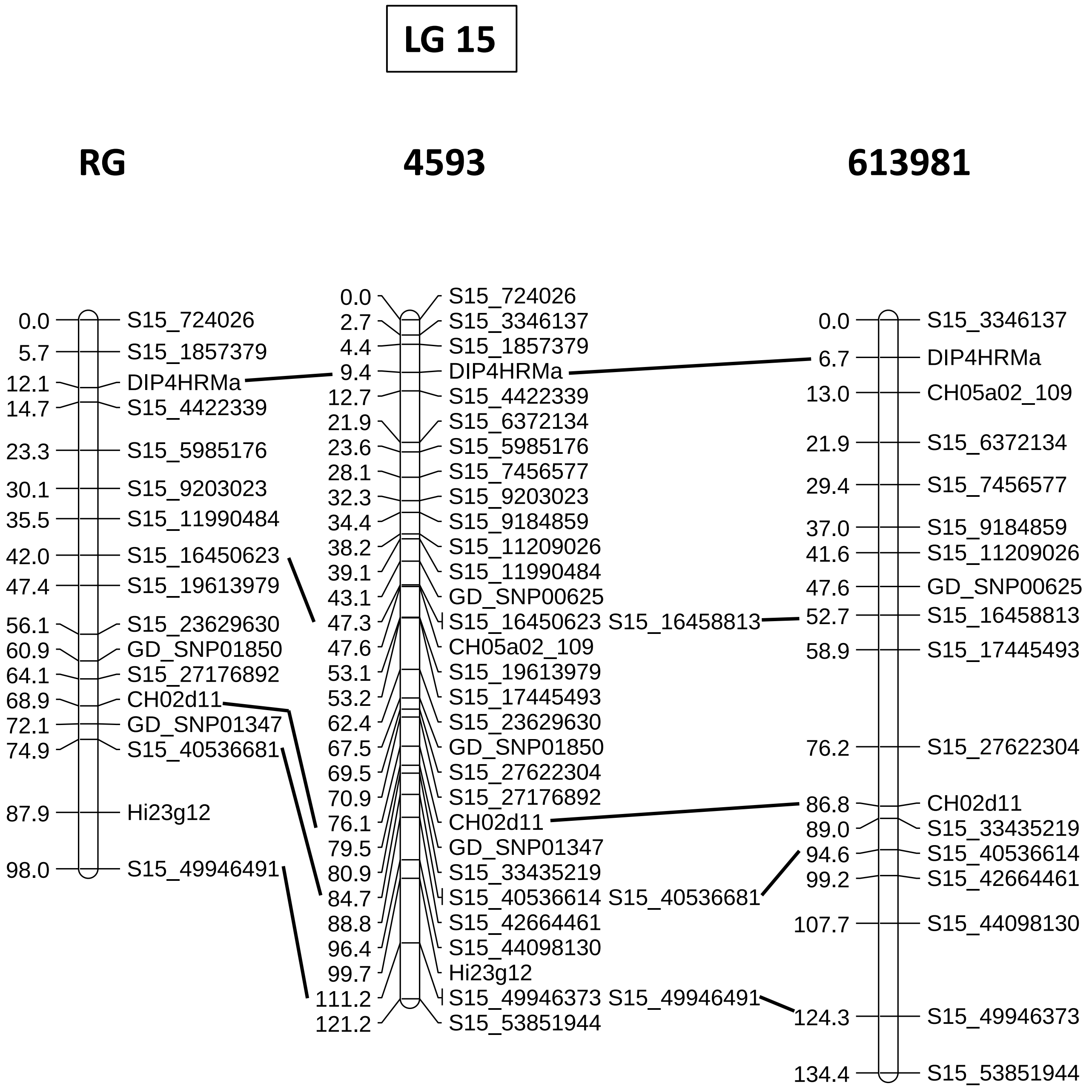
LG 15
RG
4593
613981
0.0 S15_724026
5.7 S15_1857379
12.1 DIP4HRMa
14.7 S15_4422339
23.3 S15_5985176
30.1 S15_9203023
35.5 S15_11990484
42.0 S15_16450623
47.4 S15_19613979
56.1 S15_23629630
60.9 GD_SNP01850
64.1 S15_27176892
68.9 CH02d11
72.1 GD_SNP01347
74.9 S15_40536681
87.9 Hi23g12
98.0 S15_49946491
0.0 S15_724026
2.7 S15_3346137
4.4 S15_1857379
9.4 DIP4HRMa
12.7 S15_4422339
21.9 S15_6372134
23.6 S15_5985176
28.1 S15_7456577
32.3 S15_9203023
34.4 S15_9184859
38.2 S15_11209026
39.1 S15_11990484
43.1 GD_SNP00625
47.3 S15_16450623 S15_16458813
47.6 CH05a02_109
53.1 S15_19613979
53.2 S15_17445493
62.4 S15_23629630
67.5 GD_SNP01850
69.5 S15_27622304
70.9 S15_27176892
76.1 CH02d11
79.5 GD_SNP01347
80.9 S15_33435219
84.7 S15_40536614 S15_40536681
88.8 S15_42664461
96.4 S15_44098130
99.7 Hi23g12
111.2 S15_49946373 S15_49946491
121.2 S15_53851944
0.0 S15_3346137
6.7 DIP4HRMa
13.0 CH05a02_109
21.9 S15_6372134
29.4 S15_7456577
37.0 S15_9184859
41.6 S15_11209026
47.6 GD_SNP00625
52.7 S15_16458813
58.9 S15_17445493
76.2 S15_27622304
86.8 CH02d11
89.0 S15_33435219
94.6 S15_40536614
99.2 S15_42664461
107.7 S15_44098130
124.3 S15_49946373
134.4 S15_53851944

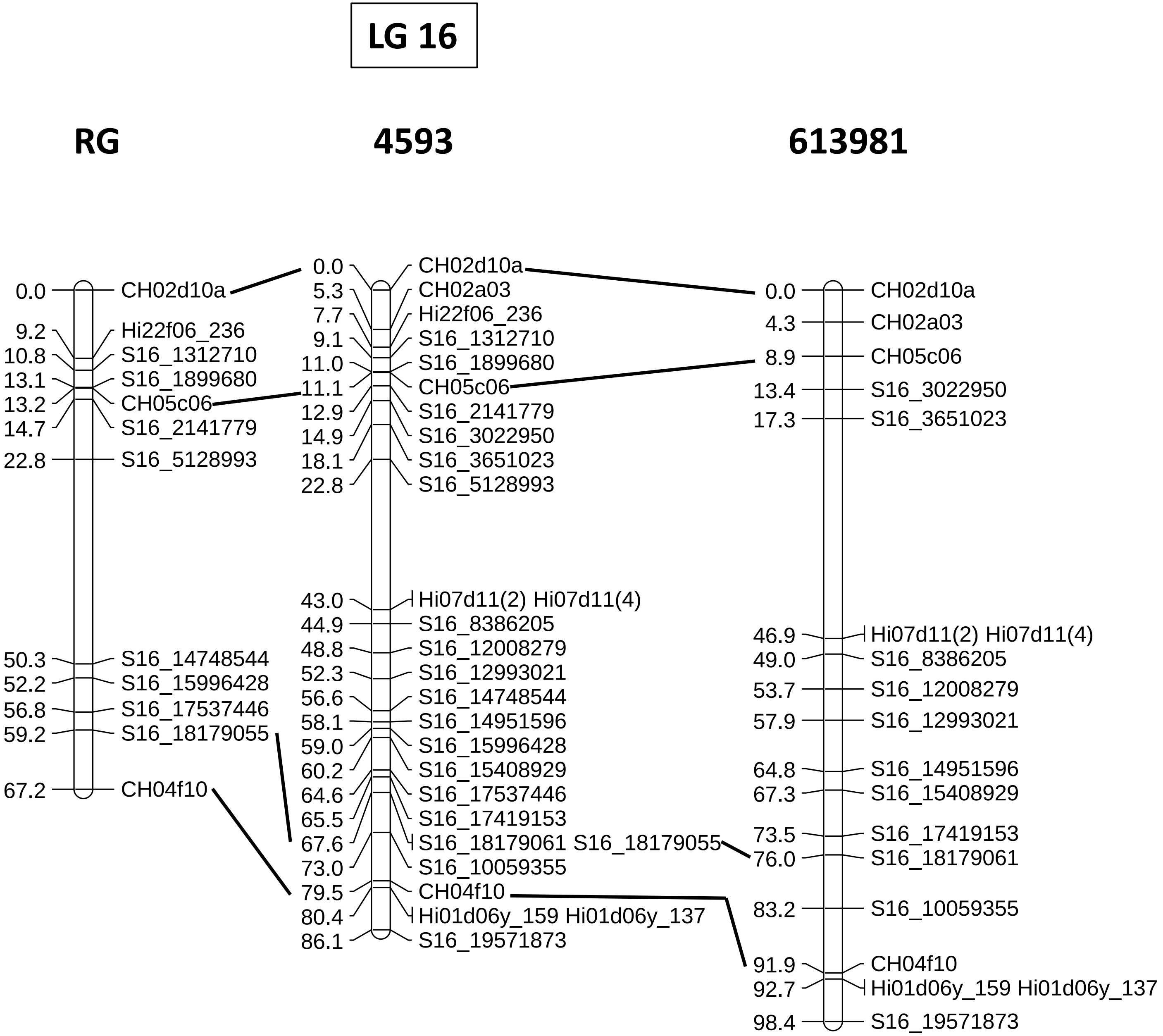

LG 16
RG
4593
613981
0.0 CH02d10a
9.2 Hi22f06_236
10.8 S16_1312710
13.1 S16_1899680
13.2 CH05c06
14.7 S16_2141779
22.8 S16_5128993
50.3 S16_14748544
52.2 S16_15996428
56.8 S16_17537446
59.2 S16_18179055
67.2 CH04f10
0.0 CH02d10a
5.3 CH02a03
7.7 Hi22f06_236
9.1 S16_1312710
11.0 S16_1899680
11.1 CH05c06
12.9 S16_2141779
14.9 S16_3022950
18.1 S16_3651023
22.8 S16_5128993
43.0 Hi07d11(2) Hi07d11(4)
44.9 S16_8386205
48.8 S16_12008279
52.3 S16_12993021
56.6 S16_14748544
58.1 S16_14951596
59.0 S16_15996428
60.2 S16_15408929
64.6 S16_17537446
65.5 S16_17419153
67.6 S16_18179061 S16_18179055
73.0 S16_10059355
79.5 CH04f10
80.4 Hi01d06y_159 Hi01d06y_137
86.1 S16_19571873
0.0 CH02d10a
4.3 CH02a03
8.9 CH05c06
13.4 S16_3022950
17.3 S16_3651023
46.9 Hi07d11(2) Hi07d11(4)
49.0 S16_8386205
53.7 S16_12008279
57.9 S16_12993021
64.8 S16_14951596
67.3 S16_15408929
73.5 S16_17419153
76.0 S16_18179061
83.2 S16_10059355
91.9 CH04f10
92.7 Hi01d06y_159 Hi01d06y_137
98.4 S16_19571873

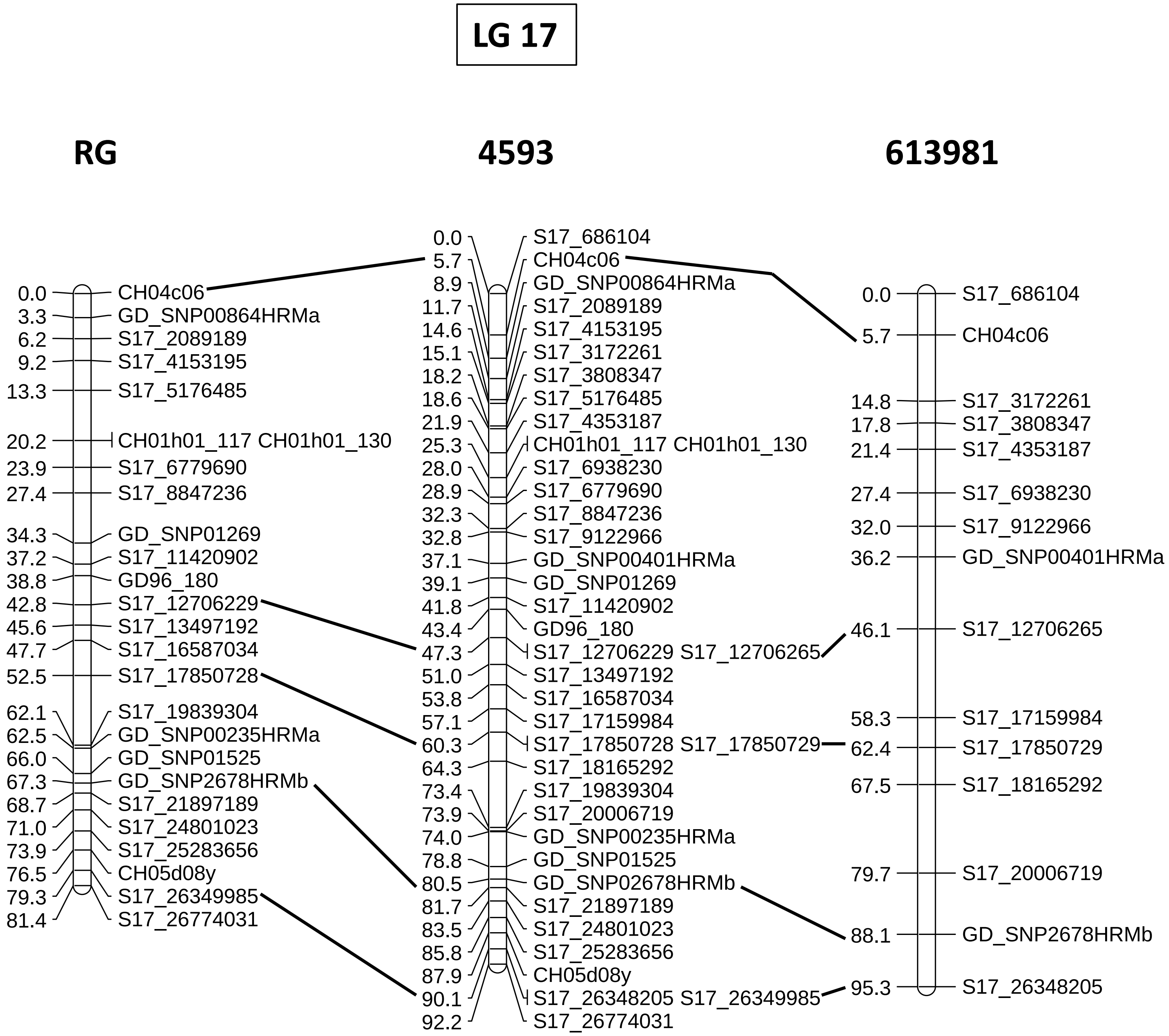

LG 17
RG
4593
613981
0.0 CH04c06
3.3 GD_SNP00864HRMa
6.2 S17_2089189
9.2 S17_4153195
13.3 S17_5176485
20.2 CH01h01_117 CH01h01_130
23.9 S17_6779690
27.4 S17_8847236
34.3 GD_SNP01269
37.2 S17_11420902
38.8 GD96_180
42.8 S17_12706229
45.6 S17_13497192
47.7 S17_16587034
52.5 S17_17850728
62.1 S17_19839304
62.5 GD_SNP00235HRMa
66.0 GD_SNP01525
67.3 GD_SNP2678HRMb
68.7 S17_21897189
71.0 S17_24801023
73.9 S17_25283656
76.5 CH05d08y
79.3 S17_26349985
81.4 S17_26774031
0.0 S17_686104
5.7 CH04c06
8.9 GD_SNP00864HRMa
11.7 S17_2089189
14.6 S17_4153195
15.1 S17_3172261
18.2 S17_3808347
18.6 S17_5176485
21.9 S17_4353187
25.3 CH01h01_117 CH01h01_130
28.0 S17_6938230
28.9 S17_6779690
32.3 S17_8847236
32.8 S17_9122966
37.1 GD_SNP00401HRMa
39.1 GD_SNP01269
41.8 S17_11420902
43.4 GD96_180
47.3 S17_12706229 S17_12706265
51.0 S17_13497192
53.8 S17_16587034
57.1 S17_17159984
60.3 S17_17850728 S17_17850729
64.3 S17_18165292
73.4 S17_19839304
73.9 S17_20006719
74.0 GD_SNP00235HRMa
78.8 GD_SNP01525
80.5 GD_SNP02678HRMb
81.7 S17_21897189
83.5 S17_24801023
85.8 S17_25283656
87.9 CH05d08y
90.1 S17_26348205 S17_26349985
92.2 S17_26774031
0.0 S17_686104
5.7 CH04c06
14.8 S17_3172261
17.8 S17_3808347
21.4 S17_4353187
27.4 S17_6938230
32.0 S17_9122966
36.2 GD_SNP00401HRMa
46.1 S17_12706265
58.3 S17_17159984
62.4 S17_17850729
67.5 S17_18165292
79.7 S17_20006719
88.1 GD_SNP2678HRMb
95.3 S17_26348205

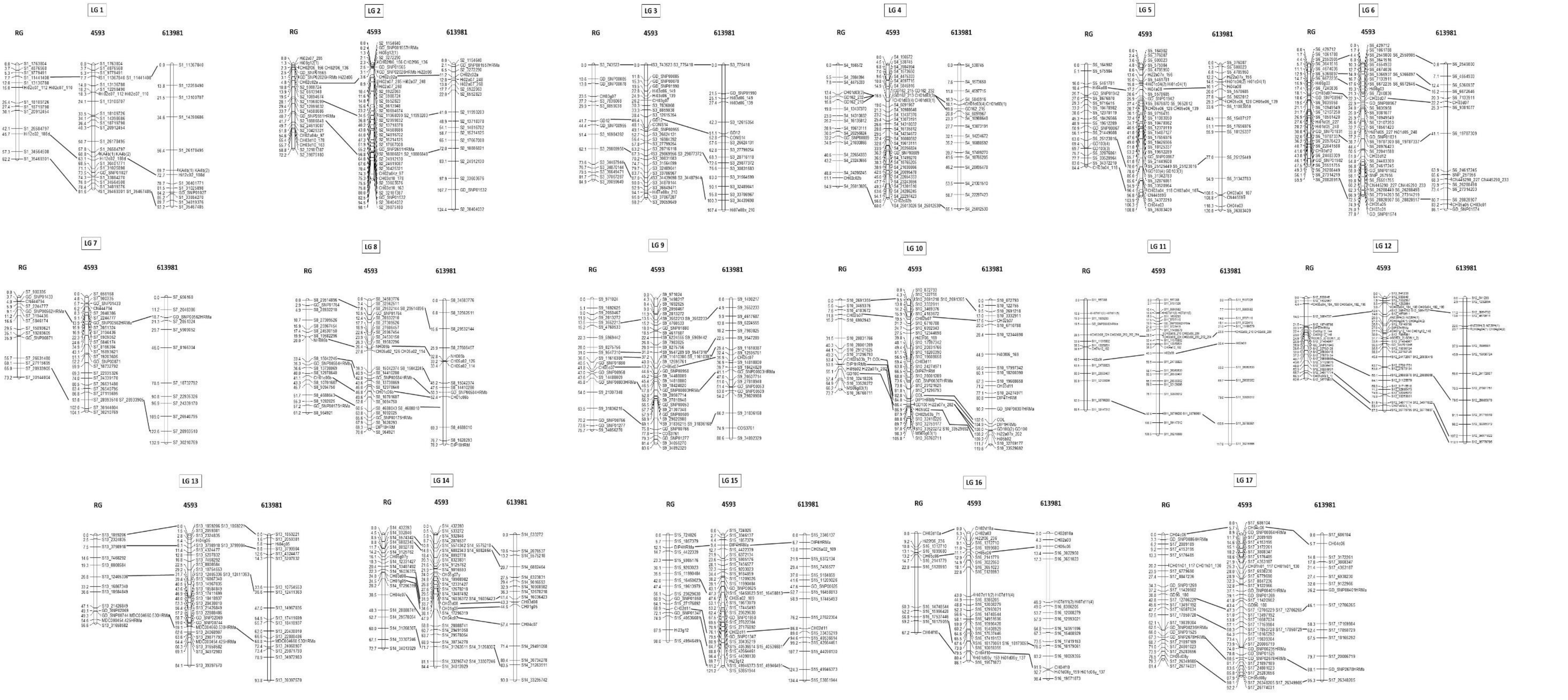
LG 1
LG 2
LG 3
LG 4
LG 5
LG 6
LG 7
LG 8
LG 9
LG 10
LG 11
LG 12
LG 13
LG 14
LG 15
LG 16
LG 17
RG
4593
613981
