# Supplementary material for: Genotyping-by-sequencing markers facilitate the identification of quantitative trait loci controlling resistance to Penicillium expansum in Malus sieversii
Source: PLoS One. 2017 Mar 3;12(3):e0172949. doi: 10.1371/journal.pone.0172949 (PMC5336245; doi:10.1371/journal.pone.0172949)

S2 Fig. ***M. sieversii*** **PI613981 marker S3_30831583 of LG3 qM-*Pe*3.1 QTL has major effect on the LSmean of blue mold lesion diameter 7 dpi.**

The parental haplotypes of *M. sieversii* PI613981 markers S3_29877372 and S3_30831583 were presumed to be *nn-np* and *np-nn*, respectively, based upon the number of individuals with each haplotypes. Both the *np-nn* parental haplotype and the *nn-nn* recombinant haplotype resulted in a 47% reduction in LSmean lesion diameter suggesting that the downstream region of the QTL is the primary contributor to resistance. Bars represent standard error of the mean.

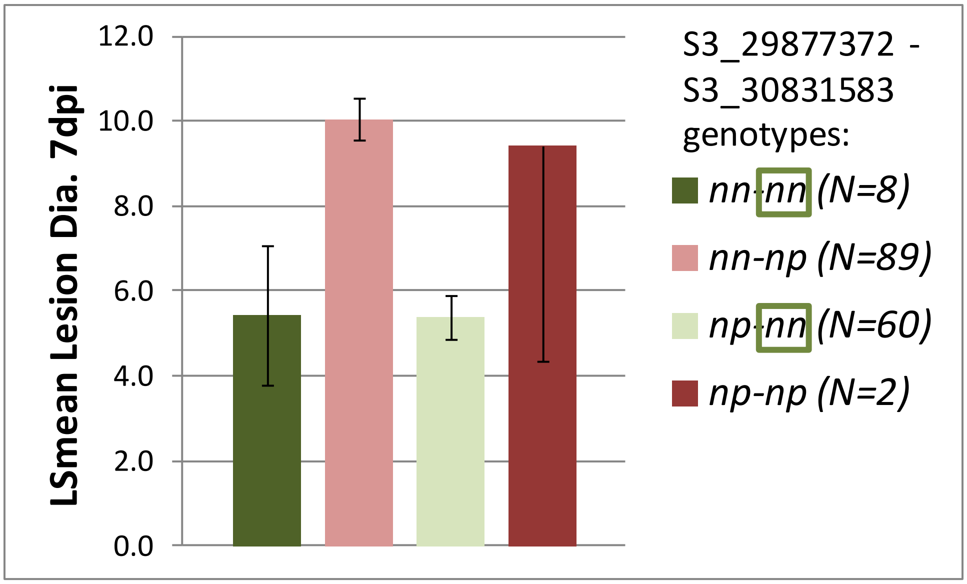

Supplement: S2 Fig — The parental haplotypes of M. sieversii PI613981 markers S3_29877372 and S3_30831583 were presumed to be nn-np and np-nn, respectively, based upon the number of individuals with each haplotypes. Both the np-nn parental haplotype and the nn-nn recombinant haplotype resulted in a 47% reduction in LSmean lesion diameter suggesting that the downstream region of the QTL is the primary contributor to resistance. Bars represent standard error of the mean. (PDF) [file pone.0172949.s002.pdf]
