# Supplementary material for: Genotyping-by-sequencing markers facilitate the identification of quantitative trait loci controlling resistance to Penicillium expansum in Malus sieversii
Source: PLoS One. 2017 Mar 3;12(3):e0172949. doi: 10.1371/journal.pone.0172949 (PMC5336245; doi:10.1371/journal.pone.0172949)

S3 Fig. Effect of LG 10 qM-*Pe*10.1 marker allele genotypes on the least square mean (LSmean) of blue mold lesion diameter 7 dpi.

A: The LSmean lesion diameter of PI613981 GDsnp00307HRM (red tones) and 'Royal Gala' S10_29121625 (blue tones) allele genotypes indicated that the *ll* allele of S10_29121625 was the primary contributor of resistance to qM-*Pe*10.1.

B: The allelic combinations of these markers suggests that the alleles of both parents are acting additively on resistance since the combined positive resistance alleles (*np,ll*), combined negative alleles (*nn,lm*) and combinations of positive and negative alleles (*nn,ll* and *np,lm*) had the lowest, highest, and intermediate LSmean lesion diameters, respectively. Bars represent the standard error of the LSmean. The arrows indicate source of alleles.

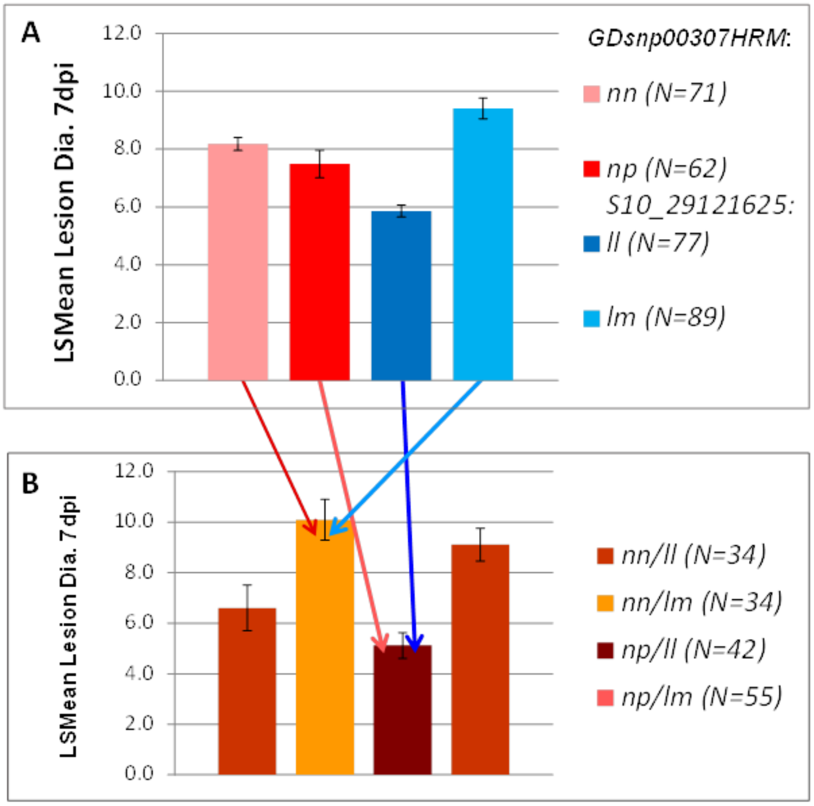

Supplement: S3 Fig — A: The LSmean lesion diameter of PI613981 GDsnp00307HRM (red tones) and ‘Royal Gala’ S10_29121625 (blue tones) allele genotypes indicated that the ll allele of S10_29121625 was the primary contributor of resistance to qM-Pe10.1. B: The allelic combinations of these markers suggests that the alleles of both parents are acting additively on resistance since the combined positive resistance alleles (np,ll), combined negative alleles (nn,lm) and combinations of positive and negative alleles (nn,ll and np,lm) had the lowest, highest, and intermediate LSmean lesion diameters, respectively. Bars represent the standard error of the LSmean. The arrows indicate source of alleles. (PDF) [file pone.0172949.s003.pdf]
