# Supplementary material for: Genotyping-by-sequencing markers facilitate the identification of quantitative trait loci controlling resistance to Penicillium expansum in Malus sieversii
Source: PLoS One. 2017 Mar 3;12(3):e0172949. doi: 10.1371/journal.pone.0172949 (PMC5336245; doi:10.1371/journal.pone.0172949)
Supplement: S1 Table — Means within a column followed by the same letter did not differ (P = 0.05) based upon a Tukey multiple comparison adjustment. Relative starch content was determined using a starch-iodine index chart for ‘Golden Delicious’ [72]. “nt” = not tested. (PDF) [file pone.0172949.s004.pdf]

**S1 Table. Overall population means of fruit quality parameters in each trial year.** Mean within a column followed by the same letter did not differ (*P*=0.05) based upon a Tukey multiple comparison adjustment. Relative starch content was determined using a starch-iodine index chart for 'Golden Delicious' (72). "nt" = not tested.

| Year | Weight (g) | Firmness (N) | Relative starch content | Soluble solids (◦ Brix) | Titrateable acidity (g $L^{-1}$ malic acid) |
|---|---|---|---|---|---|
| 2011 | 66.9 b | 94.2 cb | 5.8 a | 11.6 a | 10.1 a |
| 2012 | 39.7 a | 102.9 c | 7 a | 15.2 b | 9.9 a |
| 2013 | 60.3 b | 75.6 a | 7.7 a | 10.9 a | 9.8 a |
| 2014 | 64.7 b | 76.6 ab | 8.2 a | nt | nt |
