# Supplementary material for: Genotyping-by-sequencing markers facilitate the identification of quantitative trait loci controlling resistance to Penicillium expansum in Malus sieversii
Source: PLoS One. 2017 Mar 3;12(3):e0172949. doi: 10.1371/journal.pone.0172949 (PMC5336245; doi:10.1371/journal.pone.0172949)
Supplement: S2 Table — Source: “RG” = marker heterozygous (informative) only in ‘Royal Gala’, “Ms” = informative only in PI613981, and “Both” = informative in both parents. Mapping LOD scores for “All Years” = score calculated based on blue mold LSmean lesion diameter 7 dpi from all four years of study, whereas “No 2012” = blue mold data from drought year (2012) not included in calculation of LOD score. An “X” in “Cofactor” column indicates marker selected as cofactor in multiple QTL model analysis. (PDF) [file pone.0172949.s005.pdf]

S2 Table. QTL analysis scores for Kruskal-Wallis test, interval mapping and multiple QTL modeling on LG3 and LG10.

Linkage Group	Position (cM)	Locus	Source	Kruskal-Wallis			Interval Mapping (LOD)		Multiple QTL Model (LOD)	
				K	Df	*P=*	All Years	No 2012	No 2012	Cofactor
3	33.3	S3_8859038	RG	0.075	1	ns	1.0	1.0	0.6	
3	38.4	S3_12615354	Ms	6.262	1	0.05	1.8	1.5	0.4	
3	46.4	GD12	Both	7.879	3	0.05	2.1	2.2	0.2	
3	48.1	CONS14	Ms	12.6	1	0.0005	2.5	2.8	0.2	
3	49.1	GD_SNP00955	RG	0.316	1	ns	3.2	3.5	0.2	
3	52.7	S3_26624131	Ms	14.62	1	0.0005	4.5	5.0	0.9	
3	56.4	S3_16984392	RG	1.607	1	ns	4.2	4.6	1.1	
3	57.8	S3_27799254	Ms	13.53	1	0.0005	4.1	4.5	1.0	
3	63.2	S3_28716118	Ms	24.94	1	0.0001	6.9	7.0	0.6	
3	67.3	S3_29869956	RG	1.932	1	ns	8.1	8.5	0.7	
3	67.3	S3_29877372	Ms	29.36	1	0.0001	8.2	8.5	10.0	
3	70.2	S3_30831583	Ms	38.37	1	0.0001	11.1	11.1	13.3	X
3	74.7	S3_31564599	Ms	39.58	1	0.0001	10.4	10.9	1.0	
3	79.7	S3_32489041	Ms	16.95	1	0.0001	4.2	4.0	0.9	
3	83.7	S3_33706967	Ms	22.77	1	0.0001	5.6	5.8	0.8	
3	87	S3_34439698	Ms	11.78	1	0.001	3.5	3.8	1.1	
3	87	S3_34487944	RG	4.484	1	0.05	3.5	3.8	1.1	
10	63.8	S10_24274971	Ms	0.012	1	ns	3.2	3.2	1.4	
10	66.5	DIP47HRM	Ms	0.471	1	ns	3.2	3.4	1.0	
10	68	S10_28001289	RG	16.74	1	0.0001	3.2	3.5	0.9	
10	73.6	GD_SNP00307HRM	Ms	0.657	1	ns	4.7	5.2	7.3	
10	73.8	S10_29121625	RG	24.16	1	0.0001	4.8	5.2	7.4	X
10	81.8	S10_31296793	RG	11.85	1	0.001	3.4	3.4	1.2	
10	82.8	COL	Both	20.74	3	0.0005	3.3	3.8	0.2	
10	84	DIP1HRM	Both	17	3	0.001	3.2	3.6	0.6	
10	86.4	GD100	Both	17.78	3	0.0005	3.4	3.7	0.8	
10	86.4	Hi22a07x_202	Both	16.55	3	0.001	3.4	3.5	0.9	
10	86.9	Hi05b02	Both	15	3	0.005	3.6	3.8	1.7	
10	87.9	CH02b03b_71	RG	15.43	1	0.0001	3.5	3.8	1.5	
10	88.8	S10_32418226	RG	13.41	1	0.0005	3.8	3.9	1.0	
10	89.7	S10_32759177	Ms	2.405	1	ns	3.8	3.9	0.9	
10	97	S10_33520272	RG	6.098	1	0.05	2.3	2.4	0.3	
10	97	S10_33529692	Ms	1.862	1	0.05	2.3	2.4	0.3	
10	98.3	MS06g03(1)	RG	8.79	1	0.005	2.3	2.8	0.3	
10	105.8	S10_36760711	RG	4.557	1	0.05	2.1	2.3	0.5	

For “Source”: RG= marker heterozygous (informative) only in ‘Royal Gala, Ms= informative only in PI613981, and Both= informative in both parents. Mapping LOD scores for “All Years” = score calculated based on blue mold LSmean lesion diameter 7 dpi from all 4 years of study, whereas “No 2012” = blue mold data from drought year (2012) not included in calculation of LOD score. An “X” in “Cofactor” column indicates marker selected as cofactor in multiple QTL model analysis.
